# Assessing health system challenges and opportunities for better noncommunicable disease outcomes: the case of Mauritius

**DOI:** 10.1186/s12913-020-5039-4

**Published:** 2020-03-06

**Authors:** Laurent Musango, Maryam Timol, Premduth Burhoo, Faisal Shaikh, Philippe Donnen, Joses Muthuri Kirigia

**Affiliations:** 1 World Health Organization, Country Office for Mauritius, P.O. Box 1194, Port Louis, Mauritius; 2 Ministry of Health and Quality of Life (MOHQL), Port Louis, Mauritius; 3 Mauritius Institute of Health (MIH), Port Louis, Mauritius; 4 Ecole de Santé Publique/ULB/Brussels, Brussels, Belgium; 5 African Sustainable Development Research Consortium (ASDRC), Nairobi, Kenya

**Keywords:** Noncommunicable diseases, Population-based intervention coverage, Individual services coverage, Health system challenges

## Abstract

**Background:**

The objectives of the study reported in this paper were: (a) to score the coverage of core NCD population-based interventions and individual services in Mauritius; (b) to analyse and score the presence of 15 common health system challenges that impede delivery of core NCD interventions and services in Mauritius; and (c) to provide policy recommendations for Mauritius to address health system barriers to delivery of NCD interventions and services.

**Methods:**

The Mauritius country assessment applied the guidelines developed by the World Health Organization Regional Office for Europe for systematically scoring coverage of NCD interventions and assessing health system challenges for improving NCD outcomes. The assessment used qualitative research design approach.

**Results:**

Of the 24 core population-based interventions for addressing key NCD risk factors, 16.7% were rated extensive, 37.5% moderate and 45.8% limited. Three (20%), 8 (53%) and 4 (27%) of the 15 individual/personal CVD, diabetes and cancer services were rated extensive, moderate and limited respectively. The top five health system challenges hampering scale-up of coverage of population-based NCD interventions in Mauritius were inadequate interagency cooperation; limited application of explicit priority setting approaches; inadequate change management; sub-optimal distribution and mix human resources; insufficient population empowerment; and insufficient political commitment. The top five challenges had average scores of between 3.1 (interagency cooperation) and 2.4 (distribution and mix of human resources).

The top five health system challenges constraining expansion in coverage of individual NCD services were limited integration of evidence into practice; limited use of explicit priority-setting approaches; inadequate application of information and technology solutions; insufficient population empowerment; and sub-optimal distribution and mix of human resources. The top five challenges for individual interventions had mean scores varying between 2.6 (integration of evidence into practice) and 1.7 (distribution and mix of human resources).

**Conclusions:**

Mauritius needs to increase its domestic general government investments into the national health system and requisite multi-sectoral action to address the priority health system challenges with a view of bridging the existing gaps in coverage of NCD population-based interventions and individual services.

## Background

Mauritius is an island state in the Indian Ocean located within the continent of Africa; and had an estimated population of 1.274 million in 2018 [1]. In the same year, the country had a gross domestic product (GDP) of US$ 13.297 billion and a per capita GDP of US$ 10,437 [1]. Mauritius is an upper-middle-income economy.

Noncommunicable diseases (NCD) are the leading cause of premature mortality and disability in Mauritius. In 2016, the country lost 413,536 disability-adjusted life-years (DALY), of which 340,551 (82%) were from NCD; 43,977 (11%) from communicable, maternal, perinatal and nutritional conditions; and 29,008 (7%) from intentional and unintentional injuries [2]. Malignant neoplasms, diabetes mellitus, mental and substance use disorders, cardiovascular diseases and respiratory diseases accounted for 70.7% of NCD-related DALY loss in 2016.

According to the World Health Organization (WHO) [3], majority of NCDs emanate from four specific behaviours (harmful use of alcohol, tobacco use, physical inactivity, and unhealthy diet) that lead to four key metabolic/physiological changes (raised cholesterol, raised blood pressure, overweight/obesity and raised blood glucose). In Mauritius total pure alcohol consumption per person aged 15 years and older was 3.6 l in 2016 [4]. Age-standardized prevalence of current tobacco smoking among persons aged 15 years and older in 2015 was 21.2% [5]. The age-standardized mean population salt intake among Mauritians aged 18 years and older was 14 g per day in 2010 [6]; which was almost three times the WHO recommended daily salt intake of 5 g per person [3]. In 2016, 29.8% of adults aged 18 years and above were insufficiently physically active [7]. Modification of those behavioural risk factors requires a strong multi-sectoral action under leadership of the health sector.

The Mauritius health system infrastructure consists of 124 public health-care facilities. Of these, 88.7% are health posts, 1.61% health centres, 1.61% district hospitals, 4.03% provincial hospitals and 4.03% regional hospitals. The health post density is 8.840 per 100,000 population; 0.161 health centres per 100,000 population; 0.161 district hospitals per 100,000 population; 0.402 provincial hospitals per 100,000 population; and 0.402 regional hospitals per 100,000 population [8]. The Mauritius radiotherapy unit’s density of 2.411 per million population is higher than the average of 1.2 per million population for upper-middle-income countries but lower than the WHO European Region average of 3.9 per million population [9].

As shown in Table 1, the Mauritius health system is run by 2550 physicians, 4261 nursing and midwifery personnel, 380 dentistry personnel, 497 pharmaceutical personnel, 324 laboratory health workers, 238 environment and public health workers, 236 community and traditional health workers, 145 other health workers, and 2027 health management and support workers [10]. The Mauritius densities of health workers are lower than global averages for upper-middle-income countries [11].

**Table 1:** Numbers and densities of health workers in Mauritius

Cadres of health workers	Total number	Health workers per 1000 population in Mauritius
Physicians (2015)	2550	2.0
Nursing & midwifery personnel (2015)	4261	3.3
Dentistry personnel (2015)	380	0.3
Pharmaceutical personnel (2015)	497	0.4
Medical and Pathology Laboratory Personnel (2011)	361	0.3
Environmental and Occupational Health and Hygiene Personnel (2011)	845	0.71
Community health workers (2011)	187	0.157
Physiotherapy personnel (2011)	393	0.33
Traditional and Complementary Medicine personnel (2011)	17	0.0143

Source: WHO [10, 11]

In 2016, per capita total current health expenditure on health (CHE) in Mauritius was US$ 553 (Int$) [12]. About US$ 244 per capita came from domestic general government health expenditure; US$ 308 per capita from domestic private health expenditure; and US$ 1 per capita from external health expenditure. Mauritius CHE was within the range of US$ 297 (minimum) and US$ 984 (maximum) per person per year health systems investment recommended for achieving health sustainable development goal (SDG) 3 [13]. However, it is of concern that out-of-pocket spending (OOPS) of US$ 266 per capita, which is equivalent to 86% of private health expenditure and 48% of the total CHE, might be reducing effective financial access to health services for some people. According to the WHO World Health Report 2010 [14], when direct payments (OOPS) are above 15–20% of CHE, incidence of financial catastrophe and impoverishment increases substantially. Therefore, the OOPS in Mauritius are far much higher than the WHO threshold.

Treatment of NCDs exerts a significant burden on Mauritius health system and economy. According to Mauritius National Health Accounts 2017, of the Rupees (Rs) 25.3 billion spent on health care in 2016, Rs 16.50 billion (65.2%) was spent on treatment of NCDs. Of the total spending on NCDs, Rs 3.6 billion (21.8%) was on cardiovascular diseases, Rs 2 billion (12.1%) on respiratory diseases, Rs 1.7 billion (10.3%) on diseases of the genitourinary system, Rs 1.2 billion (7.3%) on diabetes, Rs 1.2 billion (7.3%) on mental and behavioural disorders (and neurological conditions) [15]. In addition to health system cost of managing NCDs, productivity losses associated with NCDs are significant. Stuckler, Basu and McKee [16] estimated that for every 10% increase in NCD mortality, economic growth is reduced by 0.5%. Kirigia et al. [17] estimated that NCD deaths that occurred in Mauritius in 2012 would be expected to have reduced future GDP by Int$ 1.144 billion.

The NCD-associated health and economic losses could be attributed to lack of comprehensive multi-sectoral action and suboptimal health services coverage to reduce the NCD burden. For instance, the UHC service coverage index – which encompasses population access to reproductive, maternal, new-born and child health, infectious diseases, and NCD services – was 64% [11]. This implies a service coverage gap of 36%. Prior to the assessment reported in this paper, no study had attempted to comprehensively score coverage of NCD interventions and appraise health system challenges that hamper efforts to scale-up effective coverage of NCD services in Mauritius.

The research questions of the assessment were: (a) What is the extent of implementation of the core NCD population-based interventions and individual services in Mauritius? (b) What are the common health system challenges that impede the delivery of core NCD services/interventions in Mauritius? (c) What can Mauritius do to tackle the identified health system challenges (barriers) to effective delivery of NCD interventions and services?

The objectives of the study reported in this paper were: (a) to score the coverage of core NCD population-based interventions and individual services that are essential for achievement of good NCD outcomes in Mauritius; (b) to analyse the presence of 15 common health system challenges that impede delivery of core NCD interventions and services in Mauritius; and (c) to provide policy recommendations for Mauritius to address health system barriers to delivery of NCD interventions and services.

## Methods

The current study used guidelines developed by the WHO Regional Office for Europe (EURO) to systematically assess health system challenges and opportunities for improving NCD outcomes [18]. Following the guidelines, the Mauritius country assessment team (MCAT) started with a thorough analysis of 15 years’ trends in key NCD outcome indicators derived from the WHO NCD global monitoring framework [19], details of which can be found in the detailed country assessment report [20]. The country assessment project was conducted under the overall coordination of the Acting Director General of Health Services (ADG) in the Ministry of Health and Quality of Life (MOHQL); and the WHO Country Representative (WR).

The country assessment used qualitative research design. MCAT, working groups and key informants analysed and rated (a) coverage of NCD population-based interventions and individual services; and (b) the degree of perceived hindrance that 15 common health system challenges create for interventions scale-up efforts. All the discussions were closely guided by the questions contained in the WHO assessment guide entitled “Better NCD outcomes: challenges and opportunities for health systems: Assessment Guide” [18].

We explain below the steps followed in Mauritius to achieve the three objectives of the study.

### Objective 1: scoring of the coverage of core NCD population-based interventions and individual services

#### Steps for scoring coverage of core NCD population-based interventions

The following five steps were followed to rate NCD population-based interventions used:

Step 1: Constitution of the MCAT.

The MCAT consisted of Ag Director General of Health Services, Permanent Secretary, and Director of Health Services in the Ministry of Health and Quality of Life (MOHQL); Regional Public Health Superintendent (RPHS) in Victoria Hospital; and the WHO National Consultant (team leader), Country Office Operations Officer, National Professional Officer for health promotion and NCDs, and Technical Officer for health systems. The MCAT was purposively constituted by the MOHQL.

Step 2: Review of relevance of the core population-based NCD interventions aimed at preventing tobacco consumption, preventing harmful use of alcohol, and improving diet and physical activity. Table 2 contain the 24 core population-based interventions from the WHO Global Action Plan for the prevention and control of NCD [21] that the MCAT reviewed and deemed relevant for Mauritius.

**Table 2:** Core population-based NCD interventions and global targets

*Relevant voluntary global targets by 2025*	*Core interventions*
• 30% reduction in the prevalence of current tobacco use in persons aged 15+	Increase tobacco taxes and prices to reduce affordability
	Implement plain/standardized packaging and/or large graphic health warnings on all tobacco packages
	Enact and enforce comprehensive bans on tobacco advertising, promotion and sponsorship
	Eliminate exposure to second-hand tobacco smoke in all indoor workplaces, public places, public transport
	Implement effective mass media campaigns that educate the public about the harms of smoking /tobacco use and second hand smoke
	Provide effective and population-wide support (including brief advice, national toll-free quit line services, nicotine replacement therapy) for tobacco cessation to all those who want to quit
• At least 10% reduction in the harmful use of alcohol	Use of pricing policies on alcohol including taxes on alcohol
	Restrictions and bans on alcohol advertising and promotion
	Restrictions on the availability of alcohol in the retail sector
	Minimum purchase age regulation and enforcement
	Allowed blood alcohol level for driving
• Halt the rise in diabetes and obesity • 30% reduction in mean population intake of salt/sodium • 10% reduction in the prevalence of insufficient physical activity	Reduce salt intake and salt content
	Virtually eliminate trans-fatty acids
	Implement public awareness programmes on diet and physical activity
	Reduce free sugar intake
	Increase intake of fruit and vegetables
	Reduce marketing pressure of food and non-alcoholic beverages to children
	Promote awareness about diet and physical activity

Source: WHO [21]

Step 3: MCAT review of the three category criteria developed in the WHO [18] guide for scoring coverage of population interventions. Additional File 1 contains criteria, obtained from the WHO assessment guide [18], used for scoring coverage of population interventions.

Step 4: MCAT review of pertinent national and international literature to garner evidence of implementation of the interventions in Table 2. The key documents consulted included policy papers [22, 23], legislation [24, 25], strategic frameworks/plans [26–28], health statistics [29–31], annual reports [32–38], monitoring and evaluation reports [39–41], World Bank website [42], WHO NCD-related publications [21, 43–45], WHO website [46], WHO Global Health Observatory [47], research studies and survey reports [48–50], national health accounts (NHA) reports [15, 51], and peer-reviewed articles [52, 53].

Step 6: The MCAT plus 12 purposively selected key informants had a workshop where they rated each core population-based intervention in Table 2 on a three-point scale as limited, moderate or extensive. As explained in the WHO assessment guide, a rating of:

- Limited: implies limited activities, limited commitment to real change, unimplemented initiatives, and dearth of evidence of population behaviour change for key NCD risk factors (p.12) [18].
- Moderate: means that strategies, programmes or interventions exists, reflecting commitment, but either their design is not in line with international best practice or their implementation has been hindered, leading to limited health behaviour change (p.12) [18].
- Extensive: implies evidence of extensive commitment demonstrated through strategies, programmes and interventions in line with international best practice, good implementation track record, and evidence of desired behaviour change and outcome improvement (p. 12) [18].

Among the key informants were the MOHQL senior officials, government officials from other ministries, heads of units/sections, service providers, representatives from the private sector, nongovernmental organizations, health training institutions and professional organizations.

#### Steps for scoring coverage of core NCD individual services

Table 3 contains the core individual NCD service and relevant global targets.

**Table 3:** Core individual NCD services and global targets

***Relevant voluntary global targets by 2025***	***Core services***
• At least 50% of eligible people receive drug therapy and counselling to prevent acute myocardial infarction (AMI) and stroke • 25% reduction in the prevalence of raised blood pressure or contain the prevalence of raised blood pressure	• *CVD and diabetes – first line* ✓ Risk stratification in primary health care, including hypertension, cholesterol, diabetes and other CVD risk factors ✓ Effective detection and management of hypertension, cholesterol, and diabetes through multidrug therapy based on risk stratification ✓ Effective prevention in high-risk groups and secondary prevention after AMI, including acetylsalicylic acid • *CVD and diabetes – second line* ✓ Rapid response and secondary care interventions after AMI and stroke
	• *Diabetes* ✓ Effective detection and general follow-up ✓ Patient education and intensive glucose management ✓ Hypertension management among diabetes patients ✓ Prevention of complications (e.g. eye and foot examination)
	• *Cancer – first line* ✓ Prevention of liver cancer through hepatitis B immunization ✓ Screening for cervical cancer and treatment of precancerous lesions
	• *Cancer – second line* ✓ Vaccination against human papilloma virus as appropriate if cost-effective according to national policies ✓ Early case-finding for breast cancer and timely treatment of all stages ✓ Population-based colorectal cancer screening at age > 50 linked with timely treatment ✓ Oral cancer screening in high risk groups linked with timely treatment

Source: WHO [21]

The rating of NCD individual services in Table 3 was done by the MCAT in four steps:

Step 1: Review of the core individual services for preventing, detecting, management and follow-up of cardiovascular disease, diabetes and cancer cases. Those services were extracted from the WHO Global Action Plan for the prevention and control of NCD [21].

Step 3: Review of the three category criteria developed in the WHO [18] assessment guide for scoring coverage of individual NCD services. The detailed criteria used by the MCAT in scoring coverage of individual services is contained in the Additional File 2.

Step 4: The MCAT plus 12 key informants had a workshop where, through discussion moderated by the team leader, they rated each core individual services in Table 3 on a three-point scale as limited, moderate or extensive.

The MCAT and key informants referred to various national reports and WHO global reports to confirm their ratings of the coverage of both core NCD population-based interventions and individual services.

#### Objective 2: analyse the presence of 15 common health system challenges that impede delivery of core NCD interventions and services in Mauritius

Step 1: Constitution of five working groups (WG) by the MOHQL. Each WG had a chairperson and a report writer. Combined the working groups had 37 core members; 15 government officers co-opted from relevant government ministries when required; and representatives from five non-governmental organizations (Etoile d’Esperance, MACOSS/Mauritius Heart Foundation, APSA International, TiDiams and Link to Life). The 58 key informants, were purposively selected by overall project coordinators (i.e. ADG and WR), based on their perceived expertise in the relevant health system challenge area. The composition of the five groups (without names of individuals for confidentiality) is contained in Additional File 3**.**

Step 2: Review of the 15 common health system challenges contained in Table 4 by the five WGs in plenary sessions.

**Table 4:** Fifteen health system challenges and opportunities to improve NCD outcomes

Political commitment to NCDs	Explicit priority-setting approaches	Interagency cooperation	Population empowerment
Effective model of service delivery	Coordination across providers	Regionalization	Incentive systems
Integration of evidence into practice	Distribution and mix of human resources	Access to quality medicines	Effective management
Inadequate information solutions	Managing change	Ensuring access and financial protection	

Source: Adapted from WHO/EURO [18]

The health system challenges reviewed were extracted from the.

Step 3: Assigning of the 15 common health system challenges to the five WGs as follows:

- WG1: Political commitment to NCDs, explicit priority-setting approaches, and interagency cooperation;
- WG2: Effective model of service delivery, coordination across providers, and effective management;
- WG3: Regionalization, integration of evidence into practice, and access to quality medicines;
- WG4: Distribution and mix of human resources, adequate information solutions, incentive systems, and managing change; and
- WG5: Population empowerment; and ensuring access and financial protection.

Step 4: WGs review and adaptation of the WHO guide for assessing health system challenges and opportunities in tackling NCDs [18].

Step 5: WGs appraisal and scoring of the 15 common health system challenges hindering delivery of core NCD population and individual interventions and services that had moderate or limited coverage. The salience of the health system challenges was assessed using the following scale obtained from the WHO guide [18]:

*“1 = Minor: This challenge does not prevent delivery of core interventions and services or has been fully addressed.*

*2 = Moderate: This challenge has moderate impact on the delivery of core interventions and services. Mauritius has already found ways to address it, or has solid plans to do so.*

*3 = Major: This challenge has a large negative impact on the delivery of core interventions and services. Mauritius has been struggling to find the right solutions to address it, or the chosen paths have not worked.*

*4 = Major persistent: This is a systemic problem that is persistently on the health system reform agenda and the country has not found a sustainable implementable solution or has failed numerous times to implement it”* (p.30).

The WGs were guided by the respective set of questions and matrices in the WHO assessment guide when reviewing and evaluating available information related to NCD outcomes, interventions and services for assigned features to determine how they affect the performance of health systems in delivering primary prevention, secondary prevention and disease management, and treatment of acute events.

Each WG led by an expert group chair and under the guidance of the MCAT had at least eight working sessions (characterized by debate, dialogue and deliberation), each lasting two to three hours.

Step 6: For each challenge the scores were summed up in the last row of each matrix to tease out the most important barriers undermining delivery of core interventions and services.

Step 7: Preparation of individual WG reports: Each WG prepared a short report summarizing main findings, highlighting the key challenges, and making some initial recommendations for addressing the identified challenges.

Step 8: Peer review of WG reports: First, WG chairpersons presented their reports at a one-day stakeholder workshop held on 16 November 2017, and subsequently revised their reports incorporating suggestions and filling any information gaps. Second, the WGs made final presentations of their findings and recommendations to a high-level panel of the MOHQL and development partners which was chaired by the Acting Director-General of Health Services.

Step 9: Integration and synthesis of WGs findings: After the high-level panel, WG findings were integrated and synthesized across 15 features by a WHO national consultant, and then reviewed by the MCAT. The latter also reviewed WG rating of coverage of core interventions and individual services; reviewed scoring of health system challenges; analysed the linkages between coverage evaluation and health system challenges assessment; prioritized health system challenges that most significantly undermine coverage of core NCD interventions; consolidated the policy recommendations from WGs; and prepared the draft assessment report.

## Results

### Coverage of core population and individual NCD interventions and services in Mauritius

Table 5 summarizes the assessment team’s evaluation (on a three-point scale, extensive, moderate or limited as per criteria given in the WHO assessment guide) of 24 core population-based interventions geared towards tackling the four main risk factors for NCDs, that is tobacco smoking, harmful alcohol use, unhealthy diet and physical inactivity.

**Table 5:** Mauritius scorecard for core NCD population-based interventions

Policy option	Comments	Rating
***Antismoking interventions***		
Raise tobacco taxes	Taxes on tobacco have increased on a regular basis. Currently, tobacco excise taxes constitute 57% of retail price compared to the WHO benchmark of 70% in 2016 [44].	Moderate
Provide smoke-free environments	Comprehensive smoke-free law passed and implemented; except for demarcated smoking areas in workplaces [44, 49]. Enforcement is a problem particularly in the hospitality sector.	Moderate
Issue warnings on the dangers of tobacco and tobacco smoke	Pictorial warnings covering 65% of packet size and the old set will be replaced very soon by a new set [44].	Extensive
Implement effective mass media campaigns that educate the public about the dangers of smoking/tobacco use and second-hand smoke	Regular anti-tobacco campaigns by MOHQL [44].	Moderate
Ban tobacco advertising, promotion and sponsorship	Bans on all tobacco advertising and promotion (including at points of sale) and are well enforced [44, 54].	Extensive
Provide service for tobacco cessation to all those who want to quit	Cessation clinics in all hospitals providing counselling & nicotine therapy free to all smokers willing to quit. Cessation was the in pipeline but there has been some delay [44].	Moderate
***Interventions to prevent harmful alcohol use***		
Use pricing policies on alcohol including taxes on alcohol	Almost yearly increases on alcohol taxes follow consumer price index (CPI) and increase in wages [4].	Moderate
Restrict or ban alcohol advertising and promotion	A full, well enforced ban on alcohol advertising and promotion [4, 55].	Extensive
Restrict availability of alcohol in the retail sector	Regulations restricting hours of sale exist but there are enforcement problems. Ban on sale in educational institutions [4, 55].	Limited
Enact and enforce minimum purchase age regulation	The minimum age limit for purchase of alcohol products is 18 years but effective enforcement is problematic [4, 55].	Limited
Implement a blood alcohol limit for driving	The maximum blood alcohol concentration when driving a vehicle is set at 0.5 g/L. Regular sobriety checks are carried out and there are provisions for severe penalties to those who violate [4, 49].	Limited
Provide brief psychosocial intervention for persons with hazardous and harmful alcohol use	Pharmacotherapy, psychotherapy and counselling available in public health institutions, NGOs assist in rehabilitation of alcoholic patients [4, 49].	Limited
***Interventions to improve diet***		
Reduce salt intake and the salt content of foods	Salt intake is high (7.9 g/day), a salt reduction programme is in place since 2016 [3, 6, 25, 56].	Limited
Replace trans fats with unsaturated fats	Amendment to food Regulations 1999 on the level of industrially produced trans fatty-acids in fats and oils is underway [3, 56].	Limited
Reduce free sugar intake	Taxes introduced on sugar-sweetened beverages [3, 25, 49].	Moderate
Increase consumption of fruit and vegetables	Some initiatives exist to promote consumption and availability of fruits and vegetables [3, 28]. But affordability could be a problem.	Limited
Reduce marketing pressure of food and non-alcoholic beverages to children	Regulations for school canteens exist but enforcement is a problem [3, 25, 56].	Moderate
Raise awareness on diet	Community awareness through TV and radio spots, audio-visual materials, dedicated articles and programmes, and screening activities. Curriculum on healthy lifestyle in schools [3].	Extensive
***Interventions to promote physical activity***		
Implement communitywide public education and awareness campaign for physical activity	Measures to promote physical activity through public sensitization have been undertaken [3, 49].	Moderate
Provide physical activity counselling and referral as part of routine primary health-care services through the use of a brief intervention	Physical activity counselling and referral presently not as routine primary health-care services [3, 27, 49].	Limited
Implement whole-of-school programme that includes quality physical education,	In all schools there are physical education lessons. The school curriculum includes lessons on physical activities. Physical Education is an examinable subject at School Certificate and Higher School Certificate [3, 49].	Moderate
Provide convenient and safe access to quality public open space and adequate infrastructure to support walking and cycling	There are some initiatives taken to provide adequate infrastructure for physical activity in some regions [3, 49].	Limited
Implement multicomponent workplace physical activity programmes	Facilities exist at some workplaces [3, 49]. Little progress is noted.	Limited
Promote physical activity through organized sport groups and clubs, programmes	“Sports for All’ Strategy and Action Plan for Mauritius is forthcoming [26, 27].	Limited

The coverage of 4 (16.7%) of the interventions was rated extensive, 9 (37.5%) moderate and 11 (45.8%) limited. Out of the six anti-smoking interventions, two were rated extensive and four moderate. Out of the six interventions to prevent harmful alcohol use, one was rated extensive, one moderate and four limited. Of the six interventions to improve diet, one was rated extensive, two moderate and three limited. Of the six interventions to promote physical activity, none was rated extensive, two were rated moderate and four were rated limited. According to the assessment team’s rating, Mauritius still needs to invest more in scaling up the coverage of population NCD control interventions to the extensive level.

Table 6 encapsulates the assessment team’s evaluation (on a three-point scale, as extensive, moderate or limited based on criteria given in the WHO Assessment guide) of the 15 core individual services for controlling cardiovascular diseases (CVD), diabetes and cancer.

**Table 6:** Mauritius scorecard for individual NCD services

Policy option	Score
**CVD**	
Risk stratification in primary health care [3, 29]	**Limited**
Effective detection and management of hypertension [3, 29]	**Moderate**
Effective primary prevention in high-risk groups [3, 29]	**Extensive**
Effective secondary prevention after AMI including acetylsalicylic acid [3, 29]	**Extensive**
Rapid response and secondary care after AMI and stroke [3, 29]	**Moderate**
**Diabetes**	
Effective detection and general follow-up [3]	**Moderate**
Patient education on nutrition, physical activity and glucose management [3]	**Moderate**
Hypertension management among diabetes patients [3]	**Moderate**
Prevention of complications (that is eye and foot examinations) [3]	**Moderate**
**Cancer – first line**	
Prevention of liver cancer through hepatitis B Immunization [3, 29, 49, 57, 58]	**Extensive**
Screening for cervical cancer and treatment of precancerous lesions [3, 29, 49, 57, 58]	**Moderate**
**Cancer – second line**	
Vaccination against human papilloma virus [3, 29, 49, 57, 58]	**Moderate**
Early case-finding for breast cancer and timely treatment of all stages [3, 29, 49, 57, 58]	**Limited**
Population-based colorectal cancer screening at age > 50 linked with timely treatment [3, 29, 49, 57, 58]	**Limited**
Oral cancer screening in high-risk groups linked with timely treatment [3, 29, 49, 57, 58]	**Limited**

Three (20%), eight (53%) and four (27%) of the 15 individual NCD services were rated extensive, moderate and limited respectively. With regard to CVD, effective primary prevention in high-risk groups and secondary prevention after AMI were rated extensive; effective detection and management of hypertension, and rapid response and secondary care after AMI and stroke were rated moderate; and risk stratification in primary health care was rated limited. All the individual services for diabetes (detection and general follow-up, patient education, hypertension management and prevention of complications) were rated moderate. In the case of cancer first line services, prevention of liver cancer through hepatitis B immunization was rated extensive, and screening for cervical cancer and treatment of precancerous lesions were rated moderate. Of the four cancer second line services, only vaccination against human papilloma virus was rated extensive; with the early case-finding for breast cancer and timely treatment of all stages, population-based colorectal cancer screening, and oral cancer screening coverage rated limited.

### Health system challenges hindering scale up of core NCD interventions and services

In relation to each intervention/service, the evaluation team assessed each challenge as either 1 = minor, 2 = moderate, 3 = major, or 4 = major persistent challenge. Table 7 summarizes the average scores and ranking of the top five health system challenges (interagency cooperation, explicit priority setting approaches, managing change, distribution and mix human resources, population empowerment, and political commitment) hampering scale-up of coverage of population-based NCD interventions in Mauritius.

**Table 7:** NCD population-based interventions health system challenges total scores, average scores and ranking

Health system challenge	Total score	Average score	Number of times scored 3 or 4	Rank
Political commitment	59	2.5	12	5
Explicit priority-setting approaches	67	2.8	17	2
Interagency cooperation	74	3.1	20	1
Population empowerment	59	2.5	12	5
Effective model of service delivery	–	–	–	–
Coordination across providers	–	–	–	–
Distribution and mix of human resources	58	2.4	13	4
Access to quality medicines	1	1	0	7
Effective health service management	–	–	–	–
Adequate information solutions	51	2.1	7	–
Managing change	60	2.5	13	3
Ensuring access and financial risk protection	32	1.3	0	6
Regionalization	–	–	–	–
Integration of evidence into practice	–	–	–	–
Incentive systems	–	–	–	–

Note: ‘-‘means the challenge was analysed but not scored by the assessment working groups

The top five challenges had average scores of between 3.1 (interagency cooperation) and 2.4 (distribution and mix of human resources). Additional Files 4 details the scores pertaining to degree of challenge for NCD population-based interventions.

Table 8 presents the average scores and raking of the top five health system challenges (integration of evidence into practice, explicit priority-setting approaches, adequate information solutions, population empowerment and distribution and mix of human resources) hindering optimal expansion in coverage of individual NCD services.

**Table 8:** NCD individual services health system challenges total scores, average scores and ranking

Health system challenge	Total score	Average score	Number of times scored 3 or 4	Rank
Political commitment	21	1.6	2	7
Explicit priority-setting approaches	27	2.1	3	2
Interagency cooperation	–	–	–	–
Population empowerment	24	1.8	1	4
Effective model of service delivery	–	–	–	–
Coordination across providers	–	–	–	–
Distribution and mix of human resources	22	1.7	1	5
Access to quality medicines	14	1.1	1	10
Effective health service management	–	–	–	–
Adequate information solutions	26	2	0	3
Managing change	16	1.2	0	9
Ensuring access and financial risk protection	22	1.7	0	6
Regionalization	18	1.4	1	8
Integration of evidence into practice	34	2.6	9	1
Incentive systems	–	–	–	–

Note: ‘-‘means the challenge was analysed but not scored by the assessment working groups

The top five challenges for individual interventions had mean scores varying between 2.6 (integration of evidence into practice) and 1.7 (distribution and mix of human resources). Additional Files 5 portrays the scores pertaining to degree of challenge for individual NCD services.

The remaining part of this subsection present an analysis and scores of the 15 health system features contained in Table 4.

#### Political commitment to NCDs

The Mauritius Government’s political commitment to continually improve the level and distribution of health is expressed in Mauritius Vision 2030 [22], Government Programme 2015–2019 [59], MOHQL vision and mission statement [60] and health sector strategy 2017–2021 [26]. The MOHQL mission is to create a modern high-performing quality health system that is patient centred, accessible, equitable, efficient (uses available human, financial and physical resources without waste) and innovative (using the full potential of information and communications technology) [26]. In relation to NCDs and health promotion, Mauritius strategic objective is to reduce the burden of premature morbidity, mortality and disability associated with NCDs and their risk factors [26]. At the time the assessment was conducted, efforts were underway to update the expired action plans on tobacco control [55, nutrition [28], physical activity [27] and cancer control [57].

The Government has enacted various public health legislations targeting various NCD risk factors. For instance, the 2008 public health regulations which prohibit advertisement, sponsorship and sale and consumption of alcoholic drinks in public places [55]. Another set of public health regulations that came into force in March 2009 imposed restrictions on tobacco products; and was reinforced by the June 2018 Mauritius accession to the WHO FCTC Protocol to Eliminate Illicit Trade in Tobacco Products [61]. The Minister of Health and Quality of Life published the Government Gazette of Mauritius No. Seventy-four of 15 August 2009 entitled “Food (Sale of Food on Premises of Educational Institutions) Regulations 2009”, which specifies the types of food which may be sold on the premises of educational institutions (pre-school, primary school, secondary school or pre-vocational school) [56].

Political commitment to NCDs was rated either major or major persistent challenge for 12 population interventions and for 2 individual services. The average score for population interventions was 2.5 and 1.6 for individual services. Sustaining high-level political commitment through effective budgetary and legislative support and improved coordination of NCD activities across government agencies remains an ongoing challenge.

#### Explicit priority-setting approaches

The strategic long term direction for development plans priorities has been spelt out in the Mauritius Vision 2030. The Ministry of Finance and Economic Development (MOFED) strategic plan provides a medium term strategic direction and targets. MOFED three-year strategic budget plan establishes indicative expenditure ceilings for the ministries. The MOHQL proposed annual budget estimates, which are established on historical basis, alongside those of other ministries, are reviewed by the Budget Estimates Committee meeting chaired by the MOFED.

The current budget allocation to the MOHQL is divided into five major subheads: general, curative services, primary health care and public health, treatment and prevention of HIV and AIDS, and prevention of noncommunicable diseases and promotion of quality of life (see Table 9).

**Table 9:** Distribution of allocation of government health expenditure for 2016/17 and 2019/20 financial years (Rupees 000)

Budget Head	(a) 2016/17	(b) Percent	(c) 2017/18	(d) Percent	(e) 2018/19	(f) Percent	(g) 2019/20	(h) Percent	% increase
General	425,600	3.90%	454,700	3.89%	562,100	4.68%	571,700	4.65%	34%
Hospital & specialized services	9,090,500	83.40%	9,784,900	83.80%	9,822,700	81.75%	10,007,900	81.33%	10%
Primary health care & public health	1,168,900	10.72%	1,203,900	10.31%	1,374,800	11.44%	1,465,500	11.91%	25%
Treatment & prevention of HIV/AIDS	108,200	0.99%	115,600	0.99%	121,200	1.01%	123,600	1.00%	14%
Prevention of NCDs & promotion of quality of life	106,800	0.98%	117,900	1.01%	135,200	1.13%	137,300	1.12%	29%
TOTAL	**10,900,000**	**100%**	**11,677,000**	**100%**	**12,016,000**	**100%**	**12,306,000**	**100%**	**13%**

Sources: Republic of Mauritius [15, 51]

Between 2016/17 and 2019/20 total government expenditure increased from Rupees (Rs) 10.9 to 12.3 billion, representing a 13% increase. During the same period expenditure on prevention of NCDs and promotion of quality of life grew from Rs 106.8 million to Rs 137.3 million, accounting for a 29% increase [15, 51].

Application of explicit priority-setting approaches was rated either major or major persistent challenge for 17 population interventions and for 3 individual services. The average score for population interventions was 2.8 and 2.1 for individual services.

The use of national health accounts (NHA) to link the process of national health policy development to allocation and reallocation of resources is still in the nascent stage. Moreover, the use of cost effectiveness analysis evidence in allocation of resources has not been institutionalized. Therefore, there is no explicit mechanism for prioritizing health services and public health spending. In addition, there is absence of prioritization of the health budget with regard to burden of disease, cost-effectiveness and equity considerations.

#### Interagency cooperation

The MOHQL recognizes that multi-sectoral action and partnerships are crucial for core interventions and services to have the greatest impact on NCD outcomes; the MOHQL is forming close partnerships with other sectoral ministries and national institutions; and with UN agencies, diplomatic missions, and civil society organizations including NGOs, the media and other relevant stakeholders [26]. For example, the Ministry of Education and Human Resources, Tertiary Education and Scientific Research has been an important partner in the prevention strategies which include health education, screenings and referrals, sale of healthy food items in school canteens, human papilloma virus (HPV) vaccination, etc.; the Ministry of Social Security, National Solidarity and Environment and Sustainable Development has been a partner particularly in providing preventive, promotive, curative and rehabilitative services to older people and people with disabilities; the Ministry of Agro Industry and Food Security is also collaborating with the MOHQL to ensure food security and safety and to encourage consumers to change their eating habits; the Ministry of Youth and Sports is promoting physical activities by providing incentives for purchase of sports equipment, increasing accessibility of sports infrastructure to the general public and allocation of grants to sports clubs; the Ministry of Gender Equality, Child Development and Family Welfare is organizing regular talks and sensitization campaigns on healthy eating habits, physical activities and cancer through the network of women centres in the island. The NGOs active in the health sector expressed to the assessment team strong desire to have closer cooperation, better communication and more exchange with the MOHQL.

Interagency cooperation was rated either major or major persistent challenge for 20 population interventions. The average score for population interventions was 3.1. The assessment team deemed interagency cooperation not application for individual services in Mauritius.

The main challenges include lack of functional interagency cooperation mechanism; and dearth of synergy through joint government/NGO efforts for combatting NCDs.

#### Population empowerment

The MOHQL has developed infrastructure for planning and implementation of policies, programmes, services and activities aimed at raising health literacy among the population. Some of that infrastructure includes the Health Information Education Counselling Unit; the NCD and Health Promotion Unit; and the Primary Health Care Programme. A Health Literacy Framework was developed by MOHQL in 2013; it incorporated the strengthening of the health literacy components of the different national action plans being implemented to reduce risk factors and premature mortality as well as a strategy to guide actions to improve health literacy across the life course [62].

In addition, health promotion efforts care buttressed by pertinent health awareness raising activities of the Ministry of Social Security, National Solidarity and Environment and Sustainable Development; Ministry of Gender Equality, Child Development and Family Welfare; Ministry of Education and Human Resources, Tertiary Education and Scientific Research; Ministry of Youth and Sports; and Ministry of Agro Industry and Food Security. Four NGOs also contribute to population empowerment and protection of patient rights, including the Link to Life (focusing on breast cancer), TiDiam (focussing on diabetes), APSA (focusing on diabetic foot care) and VISA (targets tobacco use).

Population empowerment was rated either major or major persistent challenge for 12 population interventions and for one individual service. The average score for population interventions was 2.5 and 1.8 for individual services.

Despite the various strategies implemented for population empowerment, there is high prevalence of NCD risk behaviours and poor adherence to treatment plans, attributed to inadequate empowerment of population to change behaviour towards taking responsibility for their own health; lack of active engagement in decision-making processes around policy issues as well as individual treatment options/plans; lack of structured peer to peer patient support groups; high-risk population groups not adequately targeted for more tailored health promotion; and lack of explicit health literacy approach for the elderly.

#### Effective models of services delivery

Mauritius has a strong primary health care system that provides health promotion (health education, empowerment and health talks), disease prevention (health check-ups and opportunistic screening), curative services (NCD clinics and diabetologist clinics) and rehabilitation [20]. The primary health care centres are manned by community physicians, nutritionists, nurses and health care assistants, among others. In principle, patients visit a primary health care provider at a community health centre/area health centre for non-emergency needs, and if necessary, the service provider issues a referral memorandum to an hospital for specialist care.

Even though the assessment team did not rate impact of effective model of services delivery on population interventions and individual services, the assessment identified factors that undermine referral system, including patients bypassing the PHC providers and going directly to secondary or tertiary hospitals for non-complicated NCD care; lack of patient identifier leading to duplication of care and dysfunctional transition of care; many PHC centres do not have optimum physician consultation time; and dearth of diagnostic and preventive services for a significant segment of the population in prediabetes stage.

#### Coordination across providers

The coordination across providers at the different levels of care in Mauritius such as home care, PHC, and emergency care, regional and specialized hospitals is patient-focused with a referral system addressing the needs of NCD patients [20]. Multidisciplinary cooperation is effective at facility level, and patients attending PHC centres are seen by a multidisciplinary team of health professionals.

Even though the assessment team did not rate impact of coordination across providers on population interventions and individual services, the working groups identified key challenges for coordination across providers to be lack of effective interoperable patient clinical data transfer system; and inefficient functioning of PHC as a hub for general coordination of care and referral to specialists.

#### Regionalization

The overall public health-care system is well structured with three distinct levels of care, namely primary, secondary and tertiary. Effective regionalization of care has been achieved with a regional hospital and an extensive PHC network in each of the five health regions with a defined catchment population [63]. All five regional hospitals have a fully equipped cardiac unit for treatment of AMI. There are no wide variations in availability and quality of services within regions. Tertiary care hospitals are accessible within reasonable driving distance. There is also a 24-h free public emergency ambulance service manned by doctors and nurses with specialized training in emergency medicine [20].

Regionalization was rated as a major challenge for scale-up of coverage of one individual NCD service, i.e. the early case-finding for breast cancer and timely treatment of all stages one individual service. The average score for individual services was 1.4. The key issues surrounding regionalization challenge included lack of clarity in the definition of roles and responsibilities for management of NCD conditions at the different health service delivery levels; and implementation of stroke unit care.

#### Incentive systems

The public health professionals receive their salaries and allowances based on recommendations of the Pay Research Bureau (institution responsible for reviewing the pay and grading structures and conditions of service in the public sector) and these are linked to position levels, years of service and responsibilities assigned [64]. Continuing professional development (CPD) is mandatory for doctors [65] and dentists [66]. Creation of the Mauritius Institute of Health (MIH) has availed opportunities for continuing education for other health workforce cadres to develop new competencies and skills, which makes them eligible for internal promotions and career advancement [67].

Inspite of the fact that the impact of incentive systems on population interventions and individual services was not rated, the working groups assessment revealed that absence of monetary incentives linked to outstanding provision of quality NCD care at individual and institutional levels. Also, since the public health service delivery system provides free access to all core NCD population-based interventions and individual services, there are no incentives for healthy behavior change in population, better self-management for patients with chronic conditions, and adherence with the referral system.

#### Access to quality medicines

Concerning access to quality of medicines, based on the WHO concept of Essential Drugs [68], the MOHQL has developed its own medicine list covering all pharmacological classes including specialized items [69]. The list is reviewed every two to 3 years by the Drug Formulary Committee to assess its adequacy and the list approved serves as a guide for medical officers at public health facilities for prescription of medicines using their generic names and for drugs that are not on the essential list on a case-by-case basis. The Hospital Drugs Committee set up at regional level evaluates such requests and advises on the purchase of drugs needed for specific cases. In addition, monitoring of prices of pharmaceutical products is carried out by relevant authorities. So far, no cases of malpractices have been found in this respect and a National Pharmacovigilance Committee (NPC) has been set up under the aegis of MOHQL to collect and analyze data on any adverse drug reactions in relation to the prescription and use of drugs in the treatment and control of disease and reporting of suspected quality issues [59]. Public procurement of medicines is highly efficient in terms of procuring medicine at competitive prices through pooled Small Developing Island Project for procurement of priority medicines.

Access to quality medicines was rated either major or major persistent challenge for none of the population interventions and for one individual service, i.e. effective detection and general follow-up of diabetes cases. The average score for population interventions was one; and 1.1 for individual services.

The WGs identified factors accounting for sub-optimal access to quality medicines to include cumbersome administrative formalities in procurement of drugs sometimes cause delays in supply; lack of dedicated quality control laboratory to monitor the quality of drugs in the local market; NPC is not very effective; and existing inventory management sometimes is the cause of stock outs at central warehouses and health facilities.

#### Integration of evidence into practice and adequate information solutions

Research [53], surveys [49, 50] and other databases on NCDs have been useful in providing local evidence for identifying more effective actions for combating NCDs. For example, the vaccination strategy against cervical cancer for young girls which started in 2016 was finalized after studies on HPV subtype prevalence done by the Central Health Laboratory and the Mauritius National Cancer Registry (MNCR) [58, 70]. The Virtual Health Library (VHL) in Mauritius which was set up in 2015 by the MIH provides all public health professionals electronic access to scientific knowledge on health [62].

Research is complemented by other information systems solutions, for instance, the civil registration systems. Morbidity conditions and mortality causes are coded according to the 10th Revision of the WHO International Classification of Diseases [71]. The Health Statistics Report published annually also contains information on population and vital statistics, infrastructure and personnel, morbidity, mortality and the activities of almost all health services pertaining to the Republic of Mauritius. Most importantly, NCD Surveys that have been regularly carried out during the last 30 years provide trends in the prevalence of NCDs and their risk factors and measure impact of actions taken previously [29–31]. Other surveys conducted periodically or on an ad hoc basis such as surveys on nutrition [72], salt intake [73], tobacco control [74, 75], household out-of-pocket expenditure [48, 52], risky behaviours in children [76] and adolescents [77–79] also provide key information that cannot be obtained from routine sources. Annual and four-year reports are published regularly from the National Cancer Registry [58]. Since 2015, Mauritius has been conducting NHA and it has since been institutionalized [15, 51].

Integration of evidence into practice was not rated as a challenge for NCD population interventions in Mauritius. However, it was rated either as a major or major persistent challenge for nine individual NCD services. The average score for individual services was 2.6.

Adequate information solutions were rated either major or major persistent challenge for seven population interventions and for none of individual services. The average score for population interventions was 2.1 and 2 for individual services. The assessment revealed that optimal integration of evidence into practice was constrained by a number of factors, including lack of a structured process for coordinated development, reviewing (for quality assurance), updating and monitoring of the NCD disease management guidelines and protocols; absence of an action research framework for prevention and control of NCDs; and structures and mechanisms for national NCD registries have not yet been institutionalized.

#### Distribution and mix of human resources

Out of the total number of human resources for health (HRH) in Mauritius, 23.9% are physicians, 40% nursing and midwifery personnel, 4.7% pharmaceutical personnel, 3.6% dentistry personnel, 3% laboratory health workers, 2.2% environment and public health workers, 2.2% community and traditional health workers, 1.4% other health workers, and 19% management and support workers [8]. In terms of health workforce distribution by services, 2.9% are in general services, 82.3% hospital and specialized services, 13.7% primary health care and public health, 0.3% treatment and prevention of HIV and AIDS, and 0.8% prevention of NCDs and promotion of quality of health. Approximately 73% of the MOHQL budget goes into remuneration of human resources for health [15]. All medical specialists are allowed private practice 2 years after their registration with the Medical Council of Mauritius.

Distribution and mix of human resources was rated either major or major persistent challenge for 13 NCD population interventions and for one individual service. The average score for population interventions was 2.4 and 1.7 for individual services. The main HRH weaknesses relate to absence of written HRH policy and needs assessment which can provide evidence to optimize health workforce performance; and limited up-to-date in-service training for service providers with regard to CVD, diabetes prevention and NCD surveillance capacity.

#### Effective health system management

**Fig. 1:**
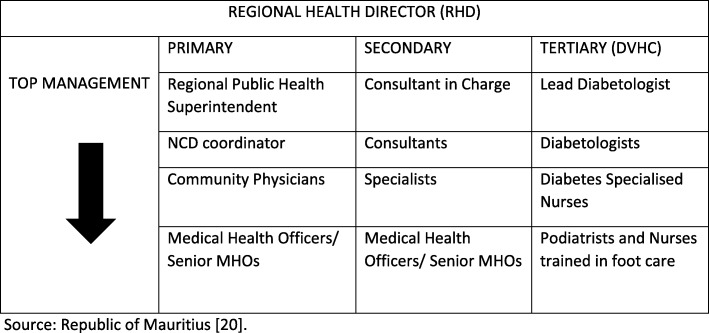
Management structure in public health system

Figure 1 shows organograms pertaining to top management positions at primary, secondary and tertiary levels of the public health system.

Regional Health Directors (RHD) are doctors with postgraduate training, appointed by the Public Service Commission (PSC) through a procedure of open competition, based on the candidates’ educational qualifications, experience and merit [20]. The authority and responsibility of managers at various levels is to ensure smooth running of concerned institutions. Directors of regional health facilities do not have decision-making power pertaining to financial resource allocation (reallocation), staffing levels, dismissal of non-performing staff, types of services to be provided, etc. Such decision is centralized at the MOHQL headquarters. Community Physicians/Senior Community Physicians and senior doctors running hospital services have all undergone management training sessions.

Despite that the impact of effective health system management on coverage of NCD population interventions and individual services was not rated, the working groups assessment revealed that existing management system of health facilities cannot effectively be used as a tool for improving quality and outcomes due to lack of standardized set of NCD clinical protocols and guidelines against which audits can be done. In addition, even though facilities have boxes for receiving patient complaints, system for receiving and analyzing patient feedback to improve patient care and outcomes is inadequate.

#### Change management

In principle entails making positive changes to attitudes, policies, systems and laws in the health sector. Mauritius has over the years introduced reforms at PHC and hospital levels with a view to improving the health services delivery, e.g.:

- the regionalization of NCD services with the creation of the posts of Community Physicians and NCD Coordinators to better manage the NCD services in the PHC system;
- the extension of opening hours of area health centres and mediclinics;
- early opening of health centres for taking of blood specimens from patients;
- introduction of staggered appointment system;
- sorting out exercises (triage) for patients attending the Accident and Emergency Departments at the regional hospitals;
- introduction of diabetologist and some other specialized clinics in PHC; and
- introduction of shift system in hospitals for medical health officers/senior medical health officers [20].

Change management was rated either major or major persistent challenge for 13 population interventions and for none of the individual services. The average score for population interventions was 2.5 and 1.2 for individual services.

The challenge of bringing about effective change in NCD prevention and control programme persists due to:

- top down approach in identifying the changes required for better health outcomes without the involvement of the health personnel and/or recipients directly concerned with the changes;
- lack of clarity of the change process, expected costs and benefits to health care providers, patients and the population;
- weak inter-sectoral collaboration mechanism;
- non-accompaniment of change by appropriate capacity building; and
- absence of regular monitoring of the change process.

#### Financial access and risk protection

In relation to access to health services and financial protection, all government health services (including diagnostic tests, medicines and follow-up) are fully tax-funded, and thus, provided for free to all users [52]. Specialized services at regional hospitals are accessible in less than 1 hour’s drive from patient’s residence. A range of free domiciliary health services are also provided to the elderly and the disabled. Furthermore, free transport facilities are provided to eligible patients upon request; and Mauritius offers free public transport to the disabled and citizens aged 60 and above. Therefore, financial burden does not currently constitute a barrier to scale-up of core NCD population interventions and individual services within the public health system.

Approximately, 73% of the population attends public health care institutions whereas 27% seek care and treatment from the private health sector on a user-fee basis [51]. OOP expenditure varied across income groups. Households earning monthly income of less than Rs20,000 spent between 7 and 12% on health compared to only 4% health spending among households earning above Rs20,000 per month [48. In 2015, 3.7% of households experienced catastrophic expenditure on health [51].

However, according to the WHO World Health Statistics 2018, Mauritius has a universal coverage index of 64%, implying that there are other factors at play that account for suboptimal coverage of essential health services (including reproductive, maternal, newborn and child health, infectious diseases, and NCD health services) [11].

Financial access and risk protection was rated either major or major persistent challenge for none of the NCD population interventions and for none of the individual services. The average score for population interventions was 1.3 and 1.7 for individual services. The main issues identified by the WGs for financial access and risk protection include: high out-of-pocket expenditure as a percentage of total health expenditure (48% of CHE); significant differences in market prices of medicines; and low health insurance coverage in the population.

## Discussion

### Key findings

The study rated coverage of core NCD population interventions and individual services; analysed the presence of 15 common health system challenges that impede delivery of core NCD interventions and services; and summarizes policy recommendations for Mauritius to address health system barriers to delivery of NCD interventions and services.

Only four (16.7%) out of the 24 core population NCD interventions and three of the 15 individual NCD services coverage was rated extensive in Mauritius. Therefore, there still remains substantial gaps in coverage of interventions aimed at reducing tobacco use, harmful alcohol use, consumption of unhealthy diet, and physical inactivity. Likewise, the individual NCD services coverage was found to be sub-optimal in the country.

Inadequate interagency cooperation and limited use of explicit priority-setting approaches were rated as major challenges to optimal expansion in coverage of population-based NCD interventions. Whereas, managing change, human resources for health, population empowerment and political commitment were other important challenges for population interventions.

Integration of evidence into practice was rated a major challenge for individual NCD services. Whereas, other health system challenges in order of priority, included use of explicit priority-setting approaches, adequate information solutions, population empowerment, distribution and mix of human resources for health, and ensuring access and financial risk protection.

### Coverage of core population NCD interventions and individual services: a comparison between Mauritius and European region countries scores

We chose to compare Mauritius and European region countries core NCD interventions coverage scores due to close epidemiological similarities. For example, 82 and 82.8% of the DALYs lost in Mauritius and the European region were from NCDs, respectively. In addition, the Mauritius life expectancy of 74.8 years was generally comparable to that of the European region of 77.5 years [2].

Additional File 6 provides a comparison of Mauritius scorecards for core population-based interventions with those of 10 European region countries, namely Belarus [80], Croatia [81], Estonia [82], Hungary [83], Kyrgyzstan [84], The former Yugoslav Republic of Macedonia [85], Moldova [86], Tajikistan [87], Turkey [88] and Serbia [89]. The European country assessments did not contain ratings for interventions to promote physical activity. Thus, comparisons are for ratings of six antismoking interventions, six interventions to prevent harmful alcohol use, and six interventions to improve diet.

#### Antismoking interventions

(a) Mauritius’s efforts to raise tobacco taxes were rated moderate; Belarus, Croatia, Macedonia, and Serbia obtained similar ratings. Only Estonia and Turkey had an extensive rating. (b) Mauritius’s efforts to combat smoking through provision of smoke-free environments were rated moderate as in Croatia, Macedonia and Tajikistan. Only Turkey had an extensive rating. (c) Issuance of warnings on the dangers of tobacco and tobacco smoke was rated extensive in Mauritius, Macedonia, Turkey, and the remaining countries had lower ratings. (d) Banning of tobacco advertising, promotion and sponsorship was rated extensive in Mauritius, Macedonia and Turkey. (e) Provision of service for tobacco cessation to all those who want to quit (nicotine replacement therapy) was assessed to be moderate in Mauritius and Estonia, and extensive in Turkey. It was only in Turkey where five of the antismoking interventions were rated extensive. There is need for WHO to undertake detailed study in Turkey with a view to documenting best practice aspects that other countries, like Mauritius, can potentially emulate to improve implementation of antismoking interventions related to raising tobacco taxes, provision of smoke-free environments, and provision of service for tobacco cessation to all those who want to quit, that is nicotine replacement therapy.

#### Interventions to prevent harmful alcohol use

(a) Use pricing policies on alcohol including taxes on alcohol was rated moderate in Kyrgyzstan and Macedonia. (b) Restriction or banning of alcohol advertising and promotion was rated extensive in Mauritius, Tajikistan and Turkey. (c) Restriction of availability of alcohol in the retail sector was rated extensive in Croatia and Turkey; and moderate in Kyrgyzstan, Macedonia and Moldova compared to limited rating in Mauritius. (d) Enactment and enforcement of minimum alcohol purchase age regulation was rated extensive in Macedonia and Turkey compared to limited rating in Mauritius. (e). Implementation of a blood alcohol limit for driving was rated extensive in Estonia and Tajikistan compared to limited rating in Mauritius.

#### Interventions to improve diet

(a) Reduction of salt intake and the salt content of foods was rated moderate/extensive only in Turkey and limited in Mauritius and all other European countries in Additional File 6. (b) Replacement of trans fats with unsaturated fats was rated moderate only in Hungary and Turkey compared to limited in Mauritius and all other European countries. (c) Reduction of free sugar intake was rated extensive in Hungary compared to moderate in Mauritius and limited in all other European countries. (d) Increased consumption of fruit and vegetables was rated limited in Mauritius compared to moderate in Belarus, Hungary, Tajikistan and Turkey. (e) Moderate rating of reduction in marketing pressure of food and non-alcoholic beverages to children in Mauritius was similar to that of Moldova and Turkey. The intervention was rated limited in other European region countries. (f) Awareness raising on diet was rated extensive in Mauritius compared to moderate in Belarus, Macedonia, Tajikistan, Turkey and Serbia.

#### Interventions to promote physical activity

(a) Implementation of communitywide public education and awareness campaigns for physical activity was similarly rated moderate in Mauritius as in Belarus, Macedonia, Tajikistan, Turkey and Serbia. (b) Provision of physical activity counselling and referral as part of routine primary health-care services through the use of a brief intervention was rated limited in Mauritius compared to moderate in Belarus. Comparison of ratings for the other interventions for promoting physical activity was not possible since information was missing for European region countries.

### Coverage of individual NCD services: a comparison between Mauritius and European region countries scores

Additional File 7 compares Mauritius scoring for coverage of individual NCD services with those of ten European region countries. The scoring information on individual cancer interventions for majority of European region countries is missing in their reports. Thus, comparisons are made only for cardiovascular diseases and diabetes individual interventions.

### Cardiovascular diseases (CVD) interventions

#### Risk stratification in primary health care

CVD risk stratification in primary health care was rated limited in Mauritius compared to moderate rating in Croatia, Kyrgyzstan, Moldova, Turkey and Serbia.

#### Effective detection and management of hypertension

Effective detection and management of hypertension was similarly rated moderate in Mauritius, Turkey and Serbia.

#### Effective primary prevention in high-risk groups

Effective CVD primary prevention in high-risk groups was rated extensive in Mauritius compared to limited/moderate in European region countries.

#### Effective secondary prevention after acute myocardial infarction (AMI) including acetylsalicylic acid

Effective secondary prevention after AMI (including acetylsalicylic acid) was similarly rated extensive in Mauritius, Macedonia, Tajikistan, Turkey and Serbia.

#### Rapid response and secondary care after AMI and stroke

Rapid response and secondary care after AMI and stroke was rated moderate in Mauritius compared to extensive in Macedonia.

### Diabetes

#### Effective detection and general follow-up

Effective detection and general follow-up for diabetes was rated moderate in Mauritius, Macedonia and Serbia.

#### Patient education on nutrition, physical activity and glucose management

Patient education on nutrition, physical activity and glucose management was rated moderate in Mauritius, Macedonia, Moldova and Serbia.

#### Hypertension management among diabetic patients

Hypertension management among people with diabetes was rated moderate in Mauritius compared to limited in Hungary, Macedonia and Serbia.

#### Prevention of complications (such as eye and foot examinations)

Prevention of diabetes complications (such as eye and foot examinations) was similarly rated moderate in Mauritius, Hungary and Serbia.

### Policy recommendations

The following recommendations are made for further development of policies, programmes and interventions to reduce exposure to NCD risk factors, improve diagnosis and treatment of NCDs, strengthen the health system and aim towards UHC in Mauritius. They can also be used as a basis for policy dialogue between the different stakeholders in the development of the Health Sector Strategic Plan and an integrated NCD action plan.

Based on the assessment of features as well as the challenges identified and discussions with key stakeholders, a number of policy recommendations emerged.

First, it was noted that current interagency cooperation is not fully functional despite a new mechanism for more effective coordination and a little synergy through joint government/NGO efforts [20]. In order to keep all stakeholders engaged in health systems strengthening for NCD outcomes, a proposal was made to establish a high-level committee (consisting of relevant ministries, the private sector, academia, NGOs and the civil society) chaired at the highest level of government that oversees and coordinates the implementation of multi-sectoral activities to better address the social determinants of health and enhance a coherent approach to Health-in-All Policies (HiAP). In addition, recommendation was made to specify mandates of each sector explicitly linked with outcomes and resources to ensure accountability; build institutional capacity; and expand health workforce competencies to address inter-sectoral agenda of NCDs and to implement core population-based interventions through whole-of-government approaches.

Second, the population (especially older people) is not adequately empowered to change behaviour towards taking responsibility for their own health and engage actively in decision-making processes both around policy issues as well as individual treatment options/plans [20]. Recommendation was made to invest in community empowerment, through health promotion approaches, to strengthen community mobilization and participation to promote health literacy for behavioural and lifestyle change; to develop incentives for disease prevention, early detection and treatment; to engage and support NGOs and patients’ groups working actively on NCDs; and to set up structured peer-to-peer support groups within different stakeholders.

Third, primary health care (PHC) in the country is inefficient contributing to weaknesses in provision of preventive services, early diagnosis and treatment for those living with NCDs [20]. In order to address this issue, it will require improving quality of health care with people-centred health services. It will entail review, update and dissemination of treatment guidelines and standards, and monitoring of compliance; shift from an acute care model to a chronic care model; auditing of clinical services at all levels of care with explicit criteria for evaluating process and outcomes**;** introduction of incentives for health workers to boost requisite capabilities for controlling NCDs; consolidating National Pharmacovigilance Committee. Furthermore, proposal was made to consolidate and scale up the role of primary health care as the centre of care for NCDs to respond to the ageing population and increasing rates of multi-morbidity. This will require strengthening PHC gatekeeping function and reducing duplication of services at PHC and hospital levels, as well as, reinforcement of the role of PHC in improving coordination between primary, secondary and tertiary care levels, implementing a more systematic screening and management of chronic conditions in PHC including improving links with NCD mobile clinics and risk stratification of patients with assessment of CVD risk factors using CVD risk scores.

Fourth, the assessment revealed issues of inadequate access, sharing and analysis of data generated by the health system, unavailability of modern information and technology solutions, and non-institutionalization of national NCD registries [20]. Recommendation was made to implement strong integrated health management information system. It will entail introducing e-health, whereby, all health information systems are integrated into an effective interoperable patient data transfer system, considering the use of a smart health card concerning all personal health information as well as setting up strong monitoring and evaluation systems.

Fifth, the assessment identified lack of explicit processes for prioritizing public health expenditures, leading to very low primary health care budgetary allocations [15]. Recommendation was made to develop and implement rational priority-setting mechanism for use in allocation of public health budget; appropriate budgeting and financing for addressing NCDs; increase substantially the allocation of funds for preventive and primary health care; earmark a fair share of the annual sin taxes collected on alcohol, tobacco and sugar for scaling up cost-effective population interventions for tackling NCD risk factors, for instance smoking, alcohol and substance abuse, unhealthy diets and low physical activity [20].

Finally, the assessment uncovered weaknesses in human resources for health (HRH) management such as the dearth of HRH planning and assessment, and inadequate NCD-related in-service training for service providers [20]. In order to improve the distribution and mix of human resources, recommendation was made to formulate a comprehensive policy and plan for HRH; improving training of HRH, especially in controlling NCDs; improving recruitment; efficient allocation of HRH; optimizing the performance of current workers via establishing clear-cut responsibilities for all grades of staff, competitive remunerations, capacity-building, performance contracts, and performance assessment; and reduction in attrition of HRH [20, 90].

### Limitations

The study had some limitations. First, as mentioned earlier, the MCAT and the WGs members were purposively chosen by the MOHQL. The key informants were also purposively selected by overall project coordinators (i.e. ADG and WR). Critiques might argue that, ideally, the MCAT, WGs and key informants should have been chosen using a statistical sampling methodology to avoid selection bias.

Second, the rating of coverage of both population-based NCD interventions and individual services was based on subjective judgements of fact by the MCAT and key informants. Thus, the ratings were dependent on the national reports and WHO reports referred to (see Tables 5 and 6), and the extent of knowledge and experience of the MCAT and key informants.

Three, the scoring of health system challenges was also based on subjective judgements of fact by the WGs and collaborated with pertinent national reports (as cited). Concerning the latter two potential limitations, like NG [91], we would argue that health scientists are more qualified than many others for making subjective judgements of fact closely related to their field study. According to NG [91], “a factual statement describes a fact as it is, and hence must be either true or false. In principle, it can be verified or falsified under ideal conditions (p.1014)”.

## Conclusion

The coverage of majority (83%) of population-based interventions aimed at combatting main NCD risk factors (tobacco smoking, harmful alcohol use, unhealthy diet and physical inactivity) was rated as either moderate or limited. The sub-optimal coverage of population-based interventions has been attributed to largely to inadequate interagency cooperation, explicit use of priority-setting approaches, change management, distribution and mix of human resources for health, population empowerment, and political commitment.

About 86.7% of the individual NCD services coverage was rated moderate or limited. The sub-optimal coverage of individual NCD services has been ascribed to insufficient integration of evidence into practice, use of explicit priority-setting approaches, use of information and technology solutions, population empowerment, and distribution and mix of human resources for health. Thus, persistent health system challenges related to explicit priority-setting approaches, population empowerment and human resource for health hinder both the optimal coverage of both population-based interventions and individual services for NCD.

Therefore, Mauritius needs to increase its domestic general government investments into the national health system and requisite multi-sectoral action to address the priority health system challenges with a view of bridging the existing gaps in coverage of NCD population-based interventions and individual services.

## Supplementary information

**Additional File 1.** Criteria used for scoring coverage of NCD population-based interventions.

**Additional File 2.** Criteria used for scoring coverage of NCD individual services.

**Additional File 3.** Composition of the five working groups members that appraised, scored and ranked common health system challenges.

**Additional File 4.** Scores pertaining to degree of health system challenge for NCD population-based interventions.

**Additional File 5.** Scores pertaining to degree of health system challenge for NCD individual services.

**Additional File 6.** Comparison of Mauritius scorecards for core population-based interventions with those of 10 European region countries.

**Additional File 7.** Comparison of Mauritius scorecards for individual NCD services with those of 10 European region countries.

## Data Availability

The NCD morbidity and mortality datasets used and/or analysed during the current study are available in the publicly accessible WHO Global Health Observatory [https://www.who.int/gho/en], the MOHQL website [http://health.govmu.org/], and MOHQL published and grey literature included in references. The data on ratings on NCD interventions and services coverage; and scores of health system challenges are included in this paper.

## Abbreviations

AMI: Acute myocardial infarction
BMI: Body mass index
CHE: Current health expenditure
CVD: Cardiovascular diseases
DALY: Disability-adjusted life-year
DBP: Diastolic blood pressure
FCTC: WHO Framework convention for Tobacco Control
GDP: Gross domestic product
HiAP: Health-in-all policies
HRH: Human resources for health
IMF: International monetary fund
Int$: International dollar or purchasing power parity
MCAT: Mauritius country assessment team
MIH: Mauritius Institute of health
MOHQL: Ministry of health and quality of life
NCD: Non-communicable diseases
NGO: Nongovernmental organization
NHA: National health accounts
OOPS: Out-of-pocket spending
PHC: Primary health care
PRB: Pay research bureau
PSC: Public service commission
RHD: Regional health director
Rs: Mauritius rupees
SBP: systolic blood pressure
SDG: UN sustainable development goals
UN: United nations
US$: United states dollar
VHL: Virtual health library
WG: working group
WHO: World health organization
WHO/EURO: WHO regional office for Europe
WR: WHO country representative
