# Supplementary material for: Assessing health system challenges and opportunities for better noncommunicable disease outcomes: the case of Mauritius
Source: BMC Health Serv Res. 2020 Mar 6;20:184. doi: 10.1186/s12913-020-5039-4 (PMC7059264; doi:10.1186/s12913-020-5039-4)
Supplement: Supplementary file 1 — Additional File 1. Criteria used for scoring coverage of NCD population-based interventions. [file 12913_2020_5039_MOESM1_ESM.docx]

**Appendix File 1: Criteria for scoring coverage of NCD population interventions**

| **Population-based interventions** | **Criteria for scoring** | | |
| --- | --- | --- | --- |
|  | **Limited** | **Moderate** | **Extensive** |
| ***Anti-smoking interventions*** |  |  |  |
| Raise tobacco taxes | Tax is less than 25% of retail price | Tax is between 25% and 75% of retail price | Tax is greater than 75% of retail price |
| Smoke-free environments | 100% smoke-free environment enforced in schools and hospitals only | 100% smoke-free environment enforced in hospitals, schools, universities, public transport and workplaces | 100% smoke-free environment enforced in all public places, including hospitality sector |
| Warnings of dangers of tobacco and smoke | Warning labels required on tobacco products, size not specified | Warning labels on all tobacco products at least 30% of package size (front and back) | Warning labels are greater than 50% of package size(front and back), with pictures (standardized packaging) |
| Bans on advertising, promotion, sponsorship | No ban, or ban on national TV, radio and print | Ban on direct and indirect advertising and promotion | Ban on all advertising and promotion, including at points of sale, with effective enforcement |
| Quit lines and nicotine replacement therapy (NRT)* | No quit lines or government funded cessation services, but NRT allowed and available for full pay by individuals | Quit lines, government-funded cessation services are available (possibly for payment). NRT available for full pay. | Toll-free quit lines, cessation services and NRT are available and affordable (covered at least partially) |
| ***Interventions to prevent harmful alcohol use*** |  |  |  |
| Raise taxes on alcohol | Alcohol taxes follow price index | Alcohol taxes follow price index; special taxes on products attractive to young people | Alcohol taxes follow price index and related to alcohol content; special taxes on products attractive to young people |
| Restrictions, bans on advertising and promotion | Regulatory frameworks exist to regulate content and volume of alcohol marketing | Regulatory frameworks exist to regulate content and volume of alcohol marketing including direct and indirect marketing and sponsorship | Full ban on alcohol marketing of any kind |
| Restrictions on availability of alcohol in retail sector | Regulatory frameworks on serving of alcohol in governmental and educational institutions | Regulatory frameworks on serving of alcohol in governmental institutions and ban on serving alcohol in educational institutions | All governmental and educational institutions free of alcohol |
| Minimum purchase age regulation and enforcement* | Minimum purchase age of 18 years for all alcohol products | Minimum age of 18 years for all alcohol products and effective enforcement | Minimum age of 18 years for all alcohol products and effective enforcement; loss of licence to sell alcohol if found breaking the law |
| Allowed blood alcohol level for driving* | Blood alcohol content maximum of 0.5 g/L | Blood alcohol content maximum 0.5 g/L, and zero for novice and professional drivers | Blood alcohol content maximum 0.2 g/L and zero for novice and professional drivers |
| **Interventions to improve diet and physical activity** |  |  |  |
| Reduce salt intake and salt content in foods | <10% reduction in salt intake in past 10 years | About 10% reduction in salt intake in past 10 years | >10% reduction in salt intake in past 10 years |
| Virtually eliminate trans-fatty acids from the diet | There is no evidence that transfats have been significantly reduced in the diet | Trans-fats reduced in some food categories and industry operators but not overall | Trans-fats eliminated from the food chain through government legislation and/or self-regulation |
| Reduce free sugar intake* | The aim to reduce the intake of free sugars is mentioned in policy documents but no action has been taken | The reduction of intake of free sugars by 5% is mentioned and partially achieved in food categories | The reduction of intake of free sugars by 5% is monitored with a focus on sugar-sweetened beverages |
| Increase intake of fruit and vegetables* | The aim to increase consumption of fruit and vegetables is mentioned but no monitoring data have been collected to support it. | The aim to increase consumption of fruit and vegetables is in line with the WHO/FAO recommendations of at least 400 g/day and some initiatives exist | The aim to increase consumption of fruit and vegetables is in line with the WHO/FAO recommendations of at least 400 g/ day with population initiatives, and incentives to increase availability, affordability and accessibility |
| Reduce marketing pressure of food and non-alcoholic beverages to children | Marketing of foods and beverages to children is noted as a problem but has not been translated into specific action in government-led initiatives. | WHO recommendations on marketing have been acknowledged and steps have been taken in self-regulatory approach to reduce marketing pressure on children | WHO recommendations on marketing and the Implementation Framework on Marketing followed consistently, including mechanism for monitoring |
| Promote awareness about diet and activity* | There has been no workforce development for nutrition and physical activity; nutrition and physical activity are not priority elements in primary care | Some workforce development for nutrition and physical activity; nutrition and physical activity are starting to be considered priority elements in primary care | Workforce development for nutrition and physical activity exists; nutrition and physical activity are priority elements in primary care |

Source: WHO [18]. Note: * Indicates criteria additional to those mentioned in the WHO [21].
