# Supplementary material for: Assessing health system challenges and opportunities for better noncommunicable disease outcomes: the case of Mauritius
Source: BMC Health Serv Res. 2020 Mar 6;20:184. doi: 10.1186/s12913-020-5039-4 (PMC7059264; doi:10.1186/s12913-020-5039-4)
Supplement: Supplementary file 2 — Additional File 2. Criteria used for scoring coverage of NCD individual services. [file 12913_2020_5039_MOESM2_ESM.docx]

**Appendix File 2: Criteria for scoring coverage of NCD individual services**

| **Population-based interventions** | **Criteria for scoring** | | |
| --- | --- | --- | --- |
|  | **Limited** | **Moderate** | **Extensive** |
| **CVD and diabetes** |  |  |  |
| Risk stratification in primary health care | 10-year CVD risk is documented in fewer than 30% of records of patients over 40 years of age with at least one main CVD risk factor; specific risk factors not routinely documented. | 10-year CVD risk is documented in 30-60% of records of patients over 40 years of age with at least one main CVD risk factor. Incomplete risk factor documentation or not using systematic method. | 10-year CVD risk routinely documented in more than 60% of records of patients over 40 years with at least one main CVD risk factor. Systematic method of calculation with routine documentation of specific risk factors. |
| Effective detection and management of hypertension | Fewer than 30% of estimated cases with high blood pressure are identified in primary health care, evidence-based generic antihypertensive drugs infrequently prescribed, no eff orts to address patient adherence. | 30-60% of estimated cases with high blood pressure are identified in primary health care, evidence-based antihypertensive drugs often (25-75%) prescribed, some eff orts to increase patient adherence but not systematic. | More than 60% of estimated cases with high blood pressure are identified in primary health care, evidence-based generic antihypertensive drugs routinely (>75%) prescribed; government funded systematic eff orts to increase adherence. |
| Effective primary prevention in high-risk groups | Prescribers not aware of indications for primary prophylaxis. Under 10% of patients with very high (>30%) 10-year CVD risk identified and prescribed multidrug regimens (antihypertensive, acetylsalicylic acid, and statin) for primary prophylaxis. Acetylsalicylic acid prescribed indiscriminately to all hypertensive patients. | Prescribers aware of indications for primary prevention with multidrug regimen. Low coverage (10-25%) of very high-risk patients with primary prophylaxis, or appropriate drug regimens prescribed but very low patient adherence. Acetylsalicylic acid prescribed indiscriminately to all HTN patients. | Routine prescription of multidrug regimens, including statins, for patients at very high CVD risk. Coverage of at-risk patients exceeds 25%. Evidence for good long-term patient adherence. Acetylsalicylic acid not prescribed to hypertensive patients with low or medium CVD risk. |
| Effective secondary prevention after AMI including acetylsalicylic acid | Fewer than 25% of patients after AMI receive acetylsalicylic acid, beta blockers and statins. | 25-75% of patients after AMI receive acetylsalicylic acid, beta blockers and statins. | More than 75% of patients after AMI receive acetylsalicylic acid, beta blockers and statins. |
| Rapid response and secondary care after AMI and stroke* | Fewer than 25% of those with AMI or stroke receive diagnosis and care within 6 hours of first symptoms | 25-50% of those with AMI or stroke receive diagnosis and care within 6 hours of first symptoms | More than 50% of those with AMI or stroke receive diagnosis and care within 6 hours of first symptoms |
| **Diabetes** |  |  |  |
| Effective detection and general follow-up * | Fewer than 75% of primary health care practices establish and maintain a register of all patients aged 17 or over with diabetes <25% detection/registration rate, based on estimated prevalence of type 2 diabetes in adult population. Not using evidence-based, systematic method to select asymptomatic patients for screening. | 25-75% of primary health care practices establish and maintain a register of all patients aged 17 or over with diabetes 25-50% detection/registration rate, based on estimated prevalence of type 2 diabetes in adult population. Using evidence-based, systematic method to select asymptomatic patients for screening, but limited coverage. | More than 75% of primary health care practices establish and maintain a register of all patients aged 17 or over with diabetes More than 50% detection/ registration rate based on estimated prevalence of type 2 diabetes in adult population. Using evidence-based, systematic method to select asymptomatic patients for screening with high coverage. |
| Patient education on nutrition and physical activity and glucose management | Fewer than 25% of those diagnosed with type 2 diabetes had at least 3 primary health care visits in past year. Fewer than 25% of registered diabetics receive organized dietary counselling. Primary health care has no counselling about physical activity. Fewer than 25% of registered diabetics had glycosylated haemoglobin measurement in past 12 months. | 25-75% of those diagnosed with type 2 diabetes had at least 3 primary health care visits in past year. 25-75% of registered diabetics receive organized dietary counselling. Primary health care routinely offers counselling on physical activity 25-75% of registered diabetics had glycosylated haemoglobin measurement in past 12 months. | More than 75% of those diagnosed with type 2 diabetes had at least 3 primary health care visits in past year. More than 75% of registered diabetics receive organized dietary counselling. Primary health care routinely offers counselling and options for physical activity through partnerships. More than 75% of registered diabetics had glycosylated haemoglobin measurement in past 12 months. |
| Hypertension management among diabetes patients | Fewer than 25% of registered diabetics with hypertension have achieved a blood pressure <140/90 mmHg; angiotensin-converting enzyme (ACE) inhibitors not routinely prescribed as first-line antihypertensive. | 25-75% of registered diabetics with hypertension have achieved a blood pressure <140/90 mmHg; ACE inhibitors routinely prescribed as first-line antihypertensive. | More than 75% of registered diabetics with hypertension have achieved a blood pressure <140/90 mmHg; ACE inhibitors routinely prescribed as first-line antihypertensive. |
| Preventing complication | Fewer than 25% of registered diabetics had a foot examination, eye examination (fundoscopy) and urine protein test in past 12 months | 25-75% of registered diabetics had a foot examination , eye examination (fundoscopy) and urine protein test in past 12 months | More than 75% of registered diabetics had a foot examination , eye examination (fundoscopy) and urine protein test in past 12 months |

Source: WHO [18]. Note: * Indicates criteria additional to those mentioned in the WHO [21].
