# Supplementary material for: Assessing health system challenges and opportunities for better noncommunicable disease outcomes: the case of Mauritius
Source: BMC Health Serv Res. 2020 Mar 6;20:184. doi: 10.1186/s12913-020-5039-4 (PMC7059264; doi:10.1186/s12913-020-5039-4)
Supplement: Supplementary file 3 — Additional File 3. Composition of the five working groups members that appraised, scored and ranked common health system challenges. [file 12913_2020_5039_MOESM3_ESM.docx]

**Additional File 3: Composition of the Mauritius National Assessment Team and Working Groups**

**Project Coordinators:** Acting Director General Health Services, (MOHQL) and WHO Representative **Assessment team:** Ag Director General, Health Services, MOH&QL; Permanent Secretary, MOH&QL; Director Health Services, MOH&QL; RPHS, MOH&QL; National Consultant, WHO Country Office *(Team Leader);* National Professional Officer (Operations), WHO Country Office; National Professional Officer (Health Promotion & NCDs), WHO Country Office; and Technical Officer, WHO Country Office.

**Composition of Working Groups**

**Working Group: 1**

**Core Areas**

- Political commitment to NCDs
- Explicit priority-setting approaches
- Interagency cooperation

**Chairperson**: Deputy Permanent Secretary, MOH&QL

**Members**: 1) Assistant Permanent secretary, MOH&QL

2) Regional Public Health Superintendent, Victoria Hospital

3) NCD Coordinator, Flacq Region, MOH&QL

4) Lead Analyst, Ministry of Finance and Economic Development

**Report writer**:

**Co-opted members** (*sessions on specific themes*):

**Other Ministries:**

1. Senior Analyst, Ministry and Economic Development
2. Technical Officer, Ministry of Education and Human Resources, Tertiary Education & Scientific Research
3. Ministry of Social Security, National Solidarity & Reforms Institutions
4. Assistant Director, Ministry of Youth and Sports
5. Ministry of Gender Equality, Child Development & Family Welfare
6. Ministry of Agro-Industry and Food Security

**NGOs:**

1. Etoile d’Esperance
2. MACOSS/Mauritius Heart Foundation
3. APSA International
4. Representative from TiDiams
5. Representative from Link to Life
6. ViSA (*invited but was absent*)

**Working Group: 2**

**Core Areas:**

- Effective model of service delivery
- Coordination across providers
- Effective management

**Chairperson**: Director, Health Services, MOH&QL

**Members**: 1) Consultant-in-Charge, Internal Medicine, J. Nehru Hospital, MOH&QL

2) Community Physician, Victoria hospital, MOH&QL

3) Head of Biochemistry Department, Victoria Hospital, MOH&QL

4) NCD Coordinator, Dr A. G. Jeetoo Hospital

5) Principal Pharmacist, MOH&QL

6) Deputy Director Nursing, MOHQL

7) Chief Nutritionist, MOH&QL

**New Members as and when required**

1. Specialist/Senior Specialist Radiotherapy
2. Diabetologist, DVHC
3. Senior Medical Records Officer

**Report Writer**:

**Working Group: 3**

**Core Areas:**

- Regionalisation
- Integration of evidence into practice
- Access to quality medicines

**Chairperson**: Director, Health Services, MOH&QL

**Members:** 1) Director, Laboratory Services, MOH&QL

2) Consultant-in-Charge, Internal Medicine, SSRNH, MOH&QL

3) Consultant-in-Charge, Internal Medicine, Flacq Hospital, MOH&QL

4) Assistant Permanent Secretary, MOH&QL

5) NCD Coordinator, SSRNH, MOH&QL

6) Deputy Director, Pharmaceutical Services, MOH&QL

**New Members as and when required**

1. Specialist/Senior Specialist Radiotherapy, MOH&QL
2. Diabetologist, DVHC, MOH&QL
3. Consultant-in-Charge, Cardiology, Victoria Hospital, MOH&QL
4. Officer-in-Charge/ Dr Boodhoo , Emergency Physician, SAMU, VH,
5. Consultant-in-Charge, Radiotherapy Department, MOH&QL

**Report Writer**:

**Working Group: 4**

**Core Areas:**

- Distribution and mix of human resources
- Adequate information solutions
- Incentive systems
- Managing change

**Chairperson**: Deputy Permanent Secretary, MOH&QL

**Members**: 1) NCD Coordinator, Victoria Hospital, MOH&QL

2) Manager Human Resources, MOH&QL

3) Public Health and Food Safety Inspector, MOPH£QL

4) Chief Health Statistician, MOH&QL

5) Assistant System Analyst, MOH&QL

**Report Writer**:

**New Members as and when required**

Senior Medical Records Officer

**Working Group: 5**

**Core Areas:**

- Population empowerment
- Ensuring access and Financial protection

**Chairperson**: Regional Public Health Superintendent, JNH, MOH&QL

**Members**: 1) Assistant Permanent Secretary, MOH&QL

2) NCD Coordinator, JNH, MOH&QL

3) Senior Analyst, MOH&QL

4) Health Promotion and Research Officer, MOH&QL

5 Assistant Medical Director, Ministry of Social Security,

National Solidarity & Reforms Institutions (replaced by Dr L

6) Ministry of Agro-Industry and Food Security

7) Senior Community Physician, MOH&QL

**Report Writer**:

**NGOs**

1. Representative from Etoile d’Esperance
2. Representative from MACOSS/Mauritius Heart Foundation
3. Representative from APSA International
4. Representative from TiDiams
5. Representative from Link to Life
6. ViSA (*invited but was absent*)
