# Supplementary material for: Assessing health system challenges and opportunities for better noncommunicable disease outcomes: the case of Mauritius
Source: BMC Health Serv Res. 2020 Mar 6;20:184. doi: 10.1186/s12913-020-5039-4 (PMC7059264; doi:10.1186/s12913-020-5039-4)
Supplement: Supplementary file 4 — Additional File 4. Scores pertaining to degree of health system challenge for NCD population-based interventions. [file 12913_2020_5039_MOESM4_ESM.docx]

**Additional File 4: Working groups score pertaining to degree of challenge for NCD population-based interventions**

| **NCD Population Interventions** | **Health system challenges** | | | | | | | | | | | |
| --- | --- | --- | --- | --- | --- | --- | --- | --- | --- | --- | --- | --- |
|  | **Political Commitment** | **Priority Setting** | **Interagency Cooperation** | **Pop Empowerment** | **Service delivery Model** | **Coordination across providers** | **Human resource** | **Access to Quality Medicine** | **Health services management** | **Adequate Info Solutions** | **Managing Change** | **Access and Financial burden** |
| **Anti-smoking interventions** |  |  |  |  |  |  |  |  |  |  |  |  |
| Raise tobacco taxes | 2 | 2 | 3 | 2 |  |  | 1 | - |  | 2 | 1 | 1 |
| Provide smoke-free environments | 3 | 3 | 3 | 2 |  |  | 2 | - |  | 2 | 2 | 1 |
| Issue warnings on the dangers of tobacco and tobacco smoke | 1 | 1 | 3 | 1 |  |  | 3 | - |  | 2 | 3 | 1 |
| Implement effective mass media campaigns that educate the public about the harms of smoking/tobacco use and second hand smoke | 3 | 3 | 2 | 1 |  |  | 3 | - |  | 3 | 2 | 1 |
| Ban tobacco advertising, promotion and sponsorship | 1 | 2 | 1 | 1 |  |  | 1 | - |  | 1 | 1 | 1 |
| Provide service for tobacco cessation to all those who want to quit | 2 | 2 | 3 | 2 |  |  | 1 | 1 |  | 1 | 2 | 2 |
| **Interventions to prevent harmful use of alcohol** |  |  |  |  |  |  |  |  |  |  |  |  |
| Use pricing policies on alcohol including taxes on alcohol | 4 | 4 | 3 | 1 |  |  | 1 | - |  | 1 | 1 | 1 |
| Restrict or ban alcohol advertising and promotion | 1 | 3 | 2 | 1 |  |  | 1 | - |  | 1 | 1 | 1 |
| Restrict availability of alcohol in the retail sector | 3 | 3 | 3 | 4 |  |  | 4 | - |  | 3 | 4 | 1 |
| Enact and enforce minimum purchase age regulation | 3 | 3 | 3 | 4 |  |  | 4 | - |  | 2 | 3 | 1 |
| Implement a blood alcohol limit for driving | 1 | 3 | 4 | 3 |  |  | 2 | - |  | 2 | 4 | 1 |
| Provide brief psychosocial intervention for persons with hazardous and harmful alcohol use | 3 | 3 | 4 | 2 |  |  | 3 | - |  | 2 | 3 | 2 |
| **Interventions to improve diet** |  |  |  |  |  |  |  |  |  |  |  |  |
| Reduce salt intake and the salt content of foods | 2 | 2 | 4 | 3 |  |  | 3 | - |  | 3 | 4 | 1 |
| Replace trans-fats with unsaturated fats | 1 | 2 | 3 | 4 |  |  | 3 | - |  | 3 | 4 | 1 |
| Reduce free sugar intake | 2 | 3 | 3 | 3 |  |  | 2 | - |  | 2 | 3 | 1 |
| Increase consumption of fruit and vegetables | 3 | 3 | 4 | 3 |  |  | 3 | - |  | 3 | 3 | 2 |
| Reduce marketing pressure of food and non-alcoholic beverages to children | 4 | 4 | 4 | 4 |  |  | 3 | - |  | 3 | 3 | 2 |
| Promote awareness about diet | 2 | 2 | 3 | 3 |  |  | 3 | - |  | 2 | 3 | 1 |
| **Interventions to promote physical activity** |  |  |  |  |  |  |  |  |  |  |  |  |
| Implement community wide public education and awareness campaign for physical activity | 2 | 3 | 2 | 2 |  |  | 2 | - |  | 2 | 1 | 2 |
| Provide physical activity counselling and referral as part of routine primary health care services through the use of a brief intervention | 4 | 4 | 4 | 3 |  |  | 3 | - |  | 3 | 2 | 1 |
| Implement whole-of-school programme that includes quality physical education, | 2 | 3 | 3 | 3 |  |  | 2 | - |  | 2 | 2 | 1 |
| Provide convenient and safe access to quality public open space and adequate infrastructure to support walking and cycling | 4 | 3 | 4 | 3 |  |  | 3 | - |  | 2 | 2 | 2 |
| Implement multi-component workplace physical activity programmes | 3 | 3 | 3 | 2 |  |  | 3 | - |  | 2 | 3 | 2 |
| Promote physical activity through organized sport groups and clubs, programmes | 3 | 3 | 3 | 2 |  |  | 2 | - |  | 2 | 3 | 2 |
| **Total Score** | 59 | 67 | 74 | 59 |  |  | 58 | 1 |  | 51 | 60 | 32 |
| **Average** | **2.5** | **2.8** | **3.1** | **2.5** |  |  | **2.4** | **1.0** |  | **2.1** | **2.5** | **1.3** |
| **Number of times Score 3 or 4** | **12.0** | **17.0** | **20.0** | **12.0** |  |  | **13.0** | **0.0** |  | **7.0** | **13.0** | **0.0** |
| **Remarks**: Interagency Cooperation and Priority Setting are considered **major** challenges | | | | | | | | | | | | |
| whereas Managing Change, Human Resources, Population Empowerment and Political Commitment are other **important** challenges | | | | | | | | | | | | |
| **Prioritisation of challenges (for population interventions):** | **Avge Score** | **Score 3 or 4** | **Ranking** |  |  |  |  |  |  |  |  |  |
| Interagency Cooperation | 3.1 | **20** | **1** |  |  |  |  |  |  |  |  |  |
| Priority setting | 2.8 | **17** | **2** |  |  |  |  |  |  |  |  |  |
| Managing Change | 2.5 | **13** | **3** |  |  |  |  |  |  |  |  |  |
| Human Resources | 2.4 | **13** | **4** |  |  |  |  |  |  |  |  |  |
| Population Empowerment | 2.5 | **12** | **5** |  |  |  |  |  |  |  |  |  |
| Political Commitment | 2.5 | **12** | **5** |  |  |  |  |  |  |  |  |  |
