# Supplementary material for: Assessing health system challenges and opportunities for better noncommunicable disease outcomes: the case of Mauritius
Source: BMC Health Serv Res. 2020 Mar 6;20:184. doi: 10.1186/s12913-020-5039-4 (PMC7059264; doi:10.1186/s12913-020-5039-4)
Supplement: Supplementary file 5 — Additional File 5. Scores pertaining to degree of health system challenge for NCD individual services. [file 12913_2020_5039_MOESM5_ESM.docx]

**Additional File 5: Working groups scores pertaining to degree of challenge for individual NCD services**

| **Individual NCD services** | **Health system challenges** | | | | | | | | | | | | | |
| --- | --- | --- | --- | --- | --- | --- | --- | --- | --- | --- | --- | --- | --- | --- |
|  | **Political Commitment** | **Priority Setting** | **Interagency Cooperation** | **Population Empowerment** | **Service delivery Model** | **Coordination across providers** | **Human resource** | **Regionalization** | **Integration Evidence into Practice** | **Access to Quality Medicine** | **Health service management** | **Adequate Information Solutions** | **Managing Change** | **Access and Financial Burden** |
| **CVD** |  |  |  |  |  |  |  |  |  |  |  |  |  |  |
| Risk stratification in primary health care | 3 | 3 | - | NA |  |  | 2 | 1 | 3 | 1 |  | 2 | 1 | NA |
| Effective detection and management of hypertension | 1 | 2 | - | 2 |  |  | 1 | 1 | 3 | 1 |  | 2 | 1 | 2 |
| Effective primary prevention in high risk groups | 2 | 2 | - | 2 |  |  | 1 | - | 3 | - |  | 2 | 1 | 2 |
| Effective secondary prevention after AMI including acetylsalicylic acid | 1 | 1 | - | 2 |  |  | 1 | 1 | 3 | 1 |  | 2 | 1 | 1 |
| Rapid response and secondary care after AMI and stroke | 1 | 2 | - | 2 |  |  | 1 | 2 | 3 | 1 |  | 2 | 1 | 2 |
| **Diabetes** |  |  |  |  |  |  |  |  |  |  |  |  |  |  |
| Effective detection and general follow-up | 1 | 2 | - | 2 |  |  | 2 | 2 | 2 | 3 |  | 2 | 1 | 2 |
| Patient education on nutrition physical activity and glucose management | 2 | 2 | - | 2 |  |  | 1 | 2 | 3 | - |  | 2 | 2 | 2 |
| Hypertension management among diabetes patients | 2 | 2 | - | 2 |  |  | 1 | 1 | 3 | 1 |  | 2 | 1 | 2 |
| Preventions of complications (e.g. eye and foot examinations) | 1 | 2 | - | 2 |  |  | 2 | 2 | 2 | 1 |  | 2 | 2 | 2 |
| **Cancer - first line** |  |  |  |  |  |  |  |  |  |  |  |  |  |  |
| Prevention of liver cancer through hepatitis B immunization | 1 | 1 | - | 1 |  |  | 2 | - | 1 | 2 |  | 2 | 1 | 1 |
| Screening for cervical cancer and treatment of precancerous lesions | 1 | 2 | - | 2 |  |  | 2 | 1 | 2 | 1 |  | 2 | 1 | 2 |
| **Cancer - second line** |  |  |  |  |  |  |  |  |  |  |  |  |  |  |
| Early case-finding for breast cancer and timely treatment of all stages | 2 | 3 | - | 2 |  |  | 2 | 3 | 3 | 1 | - | 2 | 1 | 2 |
| Population based colorectal cancer screening at age >50 linked with timely treatment | 3 | 3 | - | 3 |  |  | 4 | 2 | 3 | 1 | - | 2 | 2 | 2 |
| **Total score** | 21 | 27 |  | 24 | 0 | 0 | 22 | 18 | 34 | 14 |  | 26 | 16 | 22 |
| **Average** | **1.6** | **2.1** |  | **1.8** | **0.0** | **0.0** | **1.7** | **1.4** | **2.6** | **1.1** |  | **2.0** | **1.2** | **1.7** |
| **Number of scores 3 or 4** | **2.0** | **3.0** |  | **1.0** |  |  | **1.0** | **1.0** | **9.0** | **1.0** |  | **0.0** | **0.0** | **0.0** |
| Remarks: **Integration of Evidence into Practice** is considered a **major** challenge (with 9 scores 3 or 4). Other challenges in order of priority are: **Priority Setting, Adequate Information Solutions, , Population Empowerment, Access and Financial Burden and Human Resources.** | | | | | | | | | | | | | | |
| **Prioritisation of challenges (for Individual interventions):** |  | **Average Score** | **Score 3 or 4** | **Ranking** |  |  |  |  |  |  |  |  |  |  |
| Integration of evidence into practice | | 2.6 | 9 | **1** |  |  |  |  |  |  |  |  |  |  |
| Priority setting | | 2.1 | 3 | **2** |  |  |  |  |  |  |  |  |  |  |
| Adequate information solutions | | 2 | 0 | **3** |  |  |  |  |  |  |  |  |  |  |
| Population Empowerment | | 1.8 | 1 | **4** |  |  |  |  |  |  |  |  |  |  |
| Human Resources | | 1.7 | 1 | **5** |  |  |  |  |  |  |  |  |  |  |
| Access and Financial Burden | | 1.7 | 0 | **6** |  |  |  |  |  |  |  |  |  |  |
