# Supplementary material for: Assessing health system challenges and opportunities for better noncommunicable disease outcomes: the case of Mauritius
Source: BMC Health Serv Res. 2020 Mar 6;20:184. doi: 10.1186/s12913-020-5039-4 (PMC7059264; doi:10.1186/s12913-020-5039-4)
Supplement: Supplementary file 6 — Additional File 6. Comparison of Mauritius scorecards for core population-based interventions with those of 10 European region countries. [file 12913_2020_5039_MOESM6_ESM.docx]

**Additional File 6: Comparison of scorecards for coverage of core population-based interventions in Mauritius with those of 10 European region countries**

| **Policy option** | **Mauritius Rating** | **Belarus Rating [98]** | **Croatia Rating [91]** | **Estonia Rating**  **[92]** | **Hungary Rating [93]** | **Kyrgyzstan Rating [94]** | **Macedonia Rating [95]** | **Moldova Rating [96]** | **Tajikistan Rating [97]** | **Turkey**  **Rating [98]** | **Serbia Rating [99]** |
| --- | --- | --- | --- | --- | --- | --- | --- | --- | --- | --- | --- |
| Antismoking interventions |  |  |  |  |  |  |  |  |  |  |  |
| Raise tobacco taxes | Moderate | Moderate | Moderate | Extensive | - | Limited | Moderate | Limited | Limited | Extensive | Moderate |
| Provide smoke-free environments | Moderate | Limited | Moderate | Limited | - | Limited | Moderate | Limited | Moderate | Extensive | Limited |
| Issue warnings on the dangers of tobacco and tobacco smoke | Extensive | Moderate | Moderate | Moderate | - | Limited | Extensive | Moderate | Limited | Extensive | Limited |
| Implement effective mass-media campaigns that educate the public about the dangers of smoking/tobacco use and second-hand smoke | Moderate | - | - | - | - | - | -- | - | - | - | - |
| Ban tobacco advertising,  promotion and sponsorship | Extensive | Moderate | Moderate | Moderate | - | Moderate | Extensive | Limited | Moderate | Extensive | Limited |
| Provide service for tobacco cessation to all those who want to quit (nicotine replacement therapy) | Moderate | Limited | Limited | Moderate | - | Limited | Limited | Limited | Limited | Extensive | Limited |
| **Interventions to prevent harmful alcohol use** |  |  |  |  |  |  |  |  |  |  |  |
| Use pricing policies on alcohol including taxes on alcohol | Moderate | Limited | Limited | Limited | - | Moderate | Moderate | Limited | Limited | Limited | Limited |
| Restrict or ban alcohol advertising and promotion | Extensive | Limited | Moderate | Limited | - | Moderate | Moderate | Limited | Extensive | Extensive | Moderate |
| Restrict availability of alcohol in the retail sector | Limited | Limited | Extensive | Limited | - | Moderate | Moderate | Moderate | Limited | Extensive | Limited |
| Enact and enforce minimum purchase age regulation | Limited | Moderate | Moderate | Moderate | - | Limited | Extensive | Limited | Limited | Moderate/Extensive | Limited |
| Implement a blood alcohol limit for driving | Limited | Moderate | Moderate | Extensive | - | Moderate | Moderate | Limited | Extensive | Limited/Moderate | Moderate |
| Provide brief psychosocial intervention for persons with hazardous and harmful alcohol use | Limited | - | - | -- | - | Limited | - | - | Limited | - | - |
| **Interventions to improve diet** |  |  |  |  |  |  |  |  |  |  |  |
| Reduce salt intake and the salt content of foods | Limited | Limited | Limited | Limited | Limited | - | Limited | Limited | Limited | Moderate/Extensive | Limited |
| Replace trans fats with unsaturated fats | Limited | Limited | Limited | Limited | Moderate | - | Limited | Limited |  | Moderate | Limited |
| Reduce free sugar intake | Moderate | Limited | Limited | Limited | Extensive | -- | Limited | Limited | Limited | Limited | Limited |
| Increase consumption of fruit and vegetables | Limited | Moderate | Limited | Limited | Moderate | - | Moderate | Limited | Moderate | Moderate | Limited |
| Reduce marketing pressure of food and non-alcoholic beverages to children | Moderate | Limited | Limited | Limited | Limited | -- | Limited | Moderate | Limited | Moderate | Limited |
| Raise awareness on diet | Extensive | Moderate | Limited | Limited | Limited | - | Moderate |  | Moderate | Moderate | Moderate |
| **Interventions to promote physical activity** |  |  |  |  |  |  |  |  |  |  |  |
| Implement communitywide public education and awareness campaigns for physical activity | Moderate | Moderate | Limited | Limited | Limited | - | Moderate |  | Moderate | Moderate | Moderate |
| Provide physical activity counselling and referral as part of routine primary health-care services through the use of a brief intervention | Limited | Moderate | Limited | - | - | - | - | - | - | - | - |
| Implement whole-of-school programme that includes quality physical education | Moderate | - | - | -- | - | - | - | - | - | - | - |
| Provide convenient and safe access to quality public open space and adequate infrastructure to support walking and cycling | Limited | - | - | - | -- | - | - | - | - | - | - |
| Implement multicomponent workplace physical activity programmes | Limited | - | - | - | - | - | - | - | - | - | - |
| Promote physical activity through organized sport groups and clubs, programmes | Limited | - | - | - | - | -- | - | - | - | - | - |

**Note:** ‘-‘means that the intervention was not assessed or rates
