# Supplementary material for: Assessing health system challenges and opportunities for better noncommunicable disease outcomes: the case of Mauritius
Source: BMC Health Serv Res. 2020 Mar 6;20:184. doi: 10.1186/s12913-020-5039-4 (PMC7059264; doi:10.1186/s12913-020-5039-4)
Supplement: Supplementary file 7 — Additional File 7. Comparison of Mauritius scorecards for individual NCD services with those of 10 European region countries. [file 12913_2020_5039_MOESM7_ESM.docx]

**Additional File 7: Comparison of scorecards for coverage of individual NCD services in Mauritius with those of 10 European region countries**

| **Disease intervention** | **Mauritius Rating** | **Belarus Rating [90]** | **Croatia Rating [91]** | **Estonia Rating [92]** | **Hungary Rating [93]** | **Kyrgyzstan Rating [94]** | **Macedonia Rating [95]** | **Moldova Rating [96]** | **Tajikistan**  **Rating [97]** | **Turkey**  **Rating [98]** | **Serbia Rating [99]** |
| --- | --- | --- | --- | --- | --- | --- | --- | --- | --- | --- | --- |
| **Cardiovascular diseases (CVD)** |  |  |  |  |  |  |  |  |  |  |  |
| Risk stratification in primary health care | Limited | - | Moderate | - | - | Moderate | Limited | Moderate | Limited | Moderate | Moderate |
| Effective detection and management of hypertension | Moderate | - | Moderate | - | - | Limited | Limited | Limited | Limited | Moderate | Moderate |
| Effective primary prevention in high-risk groups | Extensive | -- | Moderate | - | - | Limited | Moderate | Limited | Limited | Moderate | Moderate |
| Effective secondary prevention after AMI including acetylsalicylic acid | Extensive | - | Moderate | - | - | Limited | Extensive | Moderate | Extensive | Extensive | Extensive |
| Rapid response and secondary care after AMI and stroke | Moderate | - | Moderate | - | - | Limited | Extensive | Limited | Limited | Moderate | Moderate |
|  | **Mauritius Rating** | **Belarus Rating [68]** | **Croatia Rating [69]** | **Estonia Rating [70]** | **Hungary Rating [71]** | **Kyrgyzstan Rating [72]** | **Macedonia Rating [73]** | **Moldova Rating [74]** | **Tajikistan**  **Rating [75]** | **Turkey**  **Rating [76]** | **Serbia Rating [77]** |
| **Diabetes** |  |  |  |  |  |  |  |  |  |  |  |
| Effective detection and general follow-up | Moderate | - | - | - | Limited | Limited | Moderate | Limited | - | - | Moderate |
| Patient education on nutrition, physical activity and glucose management | Moderate | - | - | - | Limited | Moderate | Moderate | Moderate | - | - | Moderate |
| Hypertension management among diabetic patients | Moderate | -- | - | - | Limited | - | Limited | - | - | - | Limited |
| Prevention of complications (such as eye and foot examinations) | Moderate | - | - | - | Moderate | - | Limited | - | - | - | Moderate |
| **Cancer – first line** |  |  |  |  |  |  |  |  |  |  |  |
| Prevention of liver cancer through hepatitis B immunization | Extensive | - | - | - | - | - | Extensive | - | - | - | Extensive |
| Screening for cervical cancer and treatment of precancerous lesions | Moderate | - | - | - | - | - | - | Limited | - | - | Moderate |
|  | **Mauritius Rating** | **Belarus Rating [68]** | **Croatia Rating [69]** | **Estonia Rating [70]** | **Hungary Rating [71]** | **Kyrgyzstan Rating [72]** | **Macedonia Rating [73]** | **Moldova Rating [74]** | **Tajikistan**  **Rating [75]** | **Turkey**  **Rating [76]** | **Serbia Rating [77]** |
| **Cancer – second line** |  |  |  |  |  |  |  |  |  |  |  |
| Vaccination against human papilloma virus | Moderate | - | - | - | - | - | - | - | - | - | Limited |
| Early case-finding for breast cancer and timely treatment of all stages | Limited | - | - | - | - | - | - | Limited | - | - | Moderate |
| Population-based colorectal cancer screening at age >50 linked with timely treatment | Limited | - | - | - | - | - | - | - | - | - | Moderate |
| Oral cancer screening in high-risk groups linked with timely treatment | Limited | - | - | - | - | - | - | - | - | - | - |

**Note:** ‘-‘means that the intervention was not assessed or rated.
